# Block Copolymers Based on Ethylene and Methacrylates Using a Combination of Catalytic Chain Transfer Polymerisation (CCTP) and Radical Polymerisation

**DOI:** 10.1002/anie.202108996

**Published:** 2021-10-22

**Authors:** Florian Baffie, Georgios Patias, Ataulla Shegiwal, Fabrice Brunel, Vincent Monteil, Ludmilla Verrieux, Lionel Perrin, David M. Haddleton, Franck D'Agosto

**Affiliations:** ^1^ Université de Lyon Université Lyon 1 CPE Lyon CNRS UMR 5128 Laboratoire CP2M Équipe PCM 69616 Villeurbanne, CEDEX France; ^2^ University of Warwick Department of Chemistry Gibbet Hill CV4 7AL Coventry UK; ^3^ Université de Lyon Université Claude Bernard Lyon 1 CPE Lyon INSA-Lyon CNRS UMR 5246 ICBMS 43 Bd du 11 Novembre 1918 69616 Villeurbanne France

**Keywords:** block copolymers, catalytic chain transfer polymerisation (CCTP), ethylene, macromonomers, methacrylate

## Abstract

Two scalable polymerisation methods are used in combination for the synthesis of ethylene and methacrylate block copolymers. ω‐Unsaturated methacrylic oligomers (MMA_n_) produced by catalytic chain transfer (co)polymerisation (CCTP) of methyl methacrylate (MMA) and methacrylic acid (MAA) are used as reagents in the radical polymerisation of ethylene (E) in dimethyl carbonate solvent under relatively mild conditions (80 bar, 70 °C). Kinetic measurements and analyses of the produced copolymers by size exclusion chromatography (SEC) and a combination of nuclear magnetic resonance (NMR) techniques indicate that MMA_n_ is involved in a degradative chain transfer process resulting in the formation of (MMA)_n_‐b‐PE block copolymers. Molecular modelling performed by DFT supports the overall reactivity scheme and observed selectivities. The effect of MMA_n_ molar mass and composition is also studied. The block copolymers were characterised by differential scanning calorimetry (DSC) and their bulk behaviour studied by SAXS/WAXS analysis.

## Graphical abstract

Catalytic chain transfer polymerisation was used to produce methacrylic macromonomers. Those were involved in a simple radical polymerisation of ethylene to form block copolymers featuring a polyethylene segment and various polymethacrylic segments.

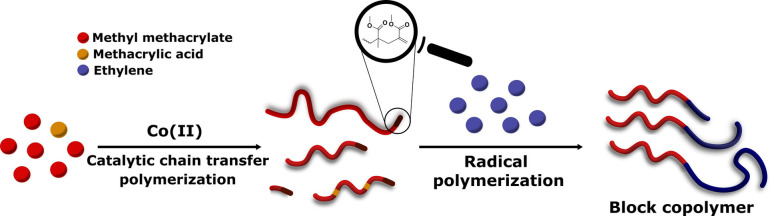

## Introduction

The ability to modify the properties of vinyl polymers by copolymerisation is well established using both statistical copolymerisation, employed widely in radical polymerisation, and block copolymerisation often employed in living polymerisation techniques.^[1]^ The ability to form block and multi‐block copolymers leads to a combination of properties which find use in numerous applications such as compatibilisers, dispersants, and surface modifiers. The desire for these types of materials is continuously increasing for the development of the next generation of high‐value polymer products for targeted properties. In order to be applied in high volume applications, both process costs and chemical costs need to be considered as well as any post polymerisation purification processes (catalyst removal). Polyethylene (PE) is the highest volume plastic produced on a global scale with only isotactic polypropylene coming close.^[2]^ Thus, PE is amongst the most important polymers/chemicals produced worldwide and the most commercially successful presenting excellent mechanical, thermal and insulating properties.^[3]^ PE is completely apolar with only C−C and C−H bonds leading to many advantages regarding stability but also providing application limitations. Even with the apparent simplicity of chemical structure, the control of branching, and subsequently crystallinity and related density, by different synthetic/production chemistry leads to many different polymers with diverse properties finding use in replacement hip joints to food packaging. However, many applications cannot be attained due to the absence of any heteroatoms. In order to increase the application of PE by improving compatibility with other fillers of polymers and to modify surface/interface properties, it is necessary to incorporate a certain degree of functionality or heteroatoms in the polymer.^[1]^ Polar–apolar block and graft copolymers can be highly valuable materials since they permit the introduction of polar moieties into the PE chain while preserving many of the original properties of the polyolefin (chemical inertia, thermal properties) in particular its ability to crystallise.^[4]^

Polyethylene is industrially produced by mostly either coordination–insertion catalytic polymerisation or radical polymerisation. While the first technique is sensitive to polar species including polar monomers that often poison the catalyst, radical polymerisation requires high temperature and pressure to (co)polymerise ethylene. Both techniques are thus incompatible with the production of complex macromolecular architectures such as polar–apolar block copolymers. Indeed, the synthesis of block copolymers based on polyolefins is challenging and often necessitates a multistep process involving distinct polymerisation techniques due to difference of reactivity between polar and apolar vinyl monomers.[^1^, ^4^, ^5^, ^6^, ^7^, ^8^] Reversible‐deactivation radical polymerisation (RDRP) techniques have become versatile methods in academia for block copolymer synthesis, mostly via sequential monomer addition.^[9]^ Radical polymerisation of ethylene under mild conditions can be challenging,^[10]^ and only recently, the controlled (co)polymerisation of ethylene with more polar monomers has been reported.[^11^, ^12^, ^13^, ^14^, ^15^, ^16^, ^17^] These discoveries have allowed for the design of more complex macromolecular architectures based on PE segments such as gradient and block copolymers.[^15^, ^18^, ^19^, ^20^, ^21^]

Although there is a significant academic interest in the use of RDRP techniques, such as atom transfer radical polymerisation (ATRP), nitroxide mediated polymerisation (NMP), organometallic mediated radical polymerisation (OMRP) or reversible addition–fragmentation chain transfer (RAFT), their use for industrial applications has been somewhat limited.[^22^, ^23^] Improvements in this area are continually being made, however, the monomer compatibility, often the presence of unwanted end‐groups in the polymeric chains, which are difficult to remove and/or change the colour of the material, the difficulty to scale up and the cost/performance ratio are some of the still existing limitations.[^24^, ^25^]

An alternative method which can overcome these limitations for polymer synthesis is cobalt(II) mediated catalytic chain transfer polymerisation (CCTP).[^26^, ^27^] The Co^II^ catalysts used in this technique are very efficient chain transfer agents in the radical polymerisation of methacrylates making this method convenient for the synthesis of low molar mass polymers with vinyl‐end functionality and even pure dimers.[^28^, ^29^, ^30^, ^31^, ^32^, ^33^, ^34^, ^35^, ^36^] The polymers/oligomers bearing a vinyl ω‐end group have been used as precursors for various materials, such as multi‐block copolymers, amphiphilic dispersants for industrial applications, block, graft and branched copolymers.[^37^, ^38^, ^39^, ^40^, ^41^]

Copolymerisation of methacrylic type ω‐unsaturated methacrylic oligomers from CCTP with methacrylates, acrylates and styrenic monomers has been studied and is reported to result in different materials.[^26^, ^42^] In the case of methacrylates, the chain transfer mechanism involves addition–fragmentation chemistry.[^40^, ^43^, ^44^] The intermediate radical formed after the addition of a propagating methacrylyl radical to the ω‐unsaturated methacrylic oligomer vinyl bond is relatively unreactive and is susceptible to fragmentation. The rate of propagation (*k*_p_) is low compared to the rate of β‐scission (fragmentation, *k*_β_), resulting in a new propagating radical and a new ω‐unsaturated methacrylic oligomer that is also capable of undergoing further addition–fragmentation chain transfer (AFCT) reaction. In the case of copolymerisation with methacrylates, β‐scission dominates leading to the formation of block copolymers by a radical route,[^40^, ^45^, ^46^, ^47^] while acrylates, *N*‐vinyl pyrrolidone, vinyl acetate, acrylonitrile and styrene have been reported to lead to the formation of graft copolymers.[^48^, ^49^] It is noted that acrylic monomers generally have higher propagation rate constants than methacrylates (15 600 L mol^−1^ s^−1^ vs. 323 L mol^−1^ s^−1^ for methyl acrylate (MA) and methyl methacrylate (MMA), respectively).[^50^, ^51^] However, some studies have shown that the copolymerisation of acrylates is unlikely to produce polymer architectures as well‐defined as earlier suggested and that polymerisation (block or graft) behaviour highly depends on the penultimate monomer unit in the ω‐unsaturated methacrylic oligomer.[^52^, ^53^, ^54^] Recently, new insights were given on the copolymerisation with acrylates by the investigation of the methacrylic macromonomers molar mass and the comonomer effects.^[37]^

Graft copolymers were obtained using low molar mass PMMA reactive oligomers and MA, whereas diblock copolymers were synthesised using higher molecular weight reactive oligomers (based on lauryl, butyl or benzyl methacrylate) or other acrylates (*n*‐butyl acrylate, BA).

Herein, the radical copolymerisation of ω‐unsaturated methacrylic oligomers, as derived from CCTP, with ethylene has been investigated. The effect of the molar mass and composition of the methacrylic oligomers on the nature of the final product is studied.

## Results and Discussion

### Synthesis of Methacrylic Oligomers via CCTP.

Polymerisation of MMA and copolymerisation of MMA with methacrylic acid (MAA) were conducted in a three‐neck, 500 mL double jacketed reactor or in a round bottom flask at 75 °C, utilising either emulsion or solution CCTP methodology.[^37^, ^47^, ^55^]

CCTP is a reliable method to synthesise methacrylic oligomers bearing an ω‐vinyl double bond and is a very efficient and scalable technique which has been used in a range of industrial applications for >25 years.[^37^, ^38^, ^47^, ^56^] In this current work, bis[(difluoroboryl) dimethylglyoximato] cobalt(II) (CoBF) was used as catalyst, as it has been proven to be a very effective chain transfer agent for the CCTP of MMA and other alkyl methacrylates.^[57]^ A range of poly(methyl methacrylate)s with different degrees of polymerisation (*n*) were prepared (MMA_*n*_, Table 1).

**Table 1:** Methacrylic oligomers synthesised by CCTP and used in this work.

MMA_*n*_	Synthesis^[a]^	Monomer^[b]^	*M*_n_ [g mol^−1^]^[c]^	*Đ* ^[c]^
MMA_2_	solution	MMA	MMA_2_ (97 %), MMA_3_ (3 %)^[d]^	–
MMA_11_	solution	MMA	1100	1.68
MMA_35_	emulsion	MMA	3500	1.85
MMA_12_MAA_2_	emulsion	MMA (85 %), MAA (15 %)	1400	1.78

[a] Polymerisation conditions used: either in solution or in emulsion, conditions are described in the experimental section. [b] Molar composition, determined by ^1^H NMR. [c] Determined by SEC. [d] Determined by GC.

### Copolymerisation of Methacrylic Dimer (MMA_2_) with Ethylene.

**Scheme 1:**
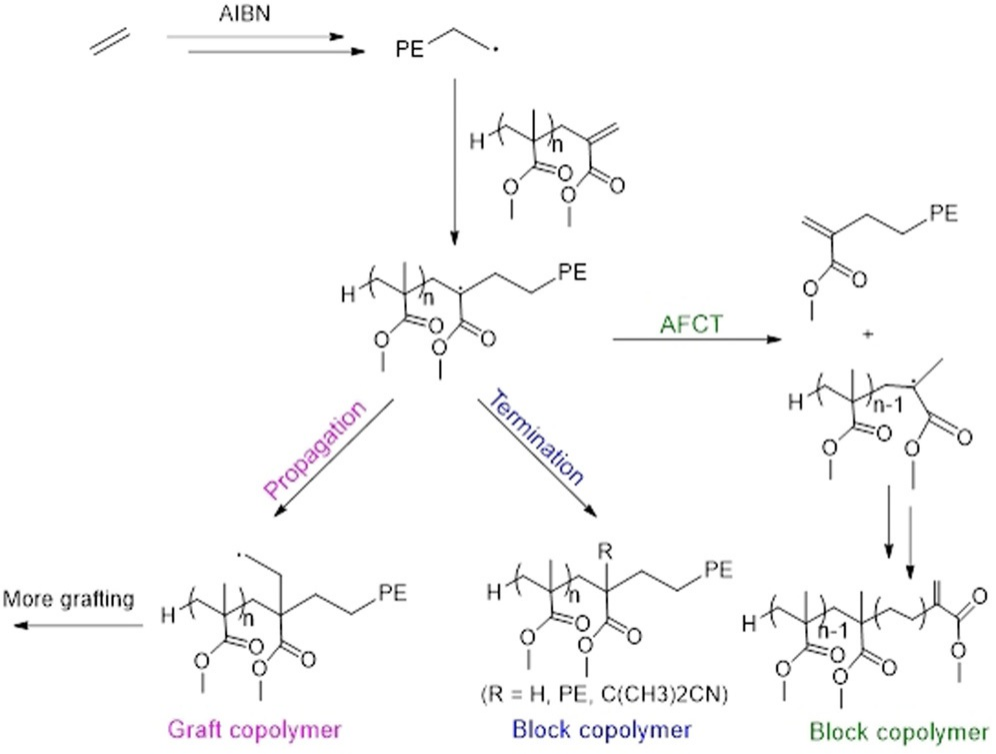
The proposed routes for the radical polymerisation of ethylene in the presence of ω‐unsaturated methacrylic oligomer leading to graft or block copolymers.

Firstly, we investigated the radical polymerisation of ethylene in the presence of MMA dimer, MMA_2_. MMA_2_ was isolated by vacuum distillation (Table S1 and Figure S1, Supporting Information). We anticipated that the analysis of the products could show either PE chains terminated with two MMA units, a “block copolymer” and/or a statistical copolymer of ethylene and MMA_2_, assimilated in a “graft polymer” which could be present with higher molar mass MMA_*n*_ (Scheme 1). Fragmentation of the intermediate radicals may also take place as might be expected in an AFCT process. Polymerisation was conducted at 70 °C and 80 bar of ethylene in 50 mL of dimethyl carbonate (DMC) as solvent with 2,2′‐azobis(isobutyronitrile) (AIBN) as initiator with a molar ratio of [MMA_2_]/[AIBN]/[ethylene]=1/1/1900. It is noted that an intermediate ethylene pressurised tank is used to charge the reactor and to maintain a constant pressure of ethylene by successive manual ethylene additions thus replenishing ethylene throughout the reaction resulting in the ratio of ethylene to MMA_2_ increasing as MMA_2_ is consumed. Figure S2 and Table 2 show the conversion of MMA_2_ as determined by GC and the consumption of ethylene as a function of polymerisation time for this system and compared with that obtained for a conventional ethylene radical polymerisation carried out under similar conditions in the absence of MMA_2_. As the reaction is conducted in an autoclave, the withdrawal of aliquots during the experiment was not possible. Therefore, each point in Figure S2 represents a different experiment. Lower yields were obtained in the presence of MMA_2_ when compared to radical homopolymerisation of ethylene. The consistent overall trends attested to the robustness and the reproducibility of the experiments. MMA_2_ was constantly consumed during the polymerisation up to completeness (Figure S2a).

**Table 2:** Radical polymerisation of ethylene in the presence of MMA_2_.

Run	*t* [h]	MMA_2_ conv.^[a]^ [%]	Ethylene cons.^[b]^ [g]	*M*_n_ (NMR)^[c]^ [g mol^−1^]	DP_PE_^[c]^	*M*_n_ (SEC)^[d]^ [g mol^−1^]	*Đ*
1^[e]^	0.8	–	0.30	–	–	7800	1.8
2^[e]^	1.5	–	0.58	–	–	7700	1.9
3^[e]^	3	–	1.15	–	–	7800	2.1
4^[e]^	6	–	2.47	–	–	8100	2.2
5	0.4	22	0.04	2050	65	2050	2.4
6	0.8	42	0.07	2350	76	2250	2.7
7	1.5	52	0.16	2400	80	2450	2.5
8	3.0	77	0.44	2650	88	2850	2.3
9	4.5	92	0.75	3450	118	3500	3.2

**Figure 1:**
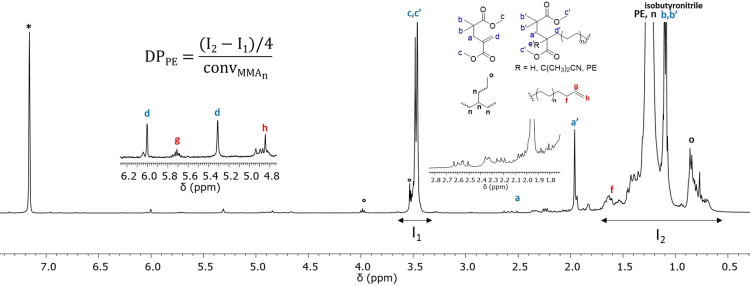
^1^H NMR spectrum (TCE/C_6_D_6_ at 90 °C) of PE synthesised in the presence of MMA_2_ (cf. Table 2 run 8). * NMR solvent benzene, ° chains initiated after transfer to polymerisation solvent (DMC). Isobutyronitrile stems from the chain‐ends of the PE initiated from AIBN.

Polymerisation conditions: AIBN (0.3 mmol), MMA_2_ (0.3 mmol) at 70 °C and 80 bar in DMC (50 mL). [a] Measured by GC. [b] Ethylene consumption=(mass of dried product)−(mass of AIBN)−(mass of MMA_2_). [c] Calculated by assuming that there is one MMA_2_ per PE chain, DP_PE_ is the degree of polymerisation and is calculated according to the equation given in Figure 1. [d] Measured by HT‐SEC using a conventional PE calibration. [e] Experiments conducted without MMA_2_.

High‐temperature SEC in trichlorobenzene (TCB) was used to estimate the molar mass of the final products. The molar masses were too low (<5000 g mol^−1^) to be measured accurately using universal calibration methodology and a conventional calibration with PE standards was preferred. The molar masses of the products obtained in presence of MMA_2_ are significantly lower than those obtained in absence of MMA_2_. Surprisingly, in presence of MMA_2_, the molar masses seem to increase with time (Figure S3 and Table 2). These results are unexpected for a conventional radical polymerisation for which the molar mass is expected to be similar all over the course of monomer conversion, as seen in the case of ethylene polymerisation (Table 2 and Figure S3c). This together with the relatively constant rate of conversion of MMA_2_ during the reaction is either consistent with an AFCT process or with MMA_2_ behaving as a “chain stopper” as opposed to a comonomer. Consequently, polyethylene chains carrying either one unsaturated chain end (AFCT) or two MMA chain end units (chain stopper) are expected to be formed. The gradual conversion of MMA_2_ would lead to the formation of higher molar mass chains upon polymerisation, which is indeed observed. In order to further understand the structure of the final polymer, additional characterisation was conducted.

^1^H NMR spectroscopy of the product formed in the presence of MMA_2_ after 3 hours was recorded at 90 °C in tetrachloroethylene (TCE)/C_6_D_6_ (2/1 by volume, Figure 1). The expected hydrogen signals characteristic for PE are observed: (i) initiation by isobutyronitrile primary radicals, from AIBN, (1.09 ppm); (ii) initiation by the radicals formed after chain transfer to solvent, DMC, (3.53 and 3.98 ppm); (iii) intramolecular and intermolecular chain transfer inherent in ethylene radical polymerisation leading to long and short‐chain branching (0.61–0.90 ppm); (iv) trace presence of vinyl end groups (4.93 and 5.76 ppm) as already observed.[^11^, ^12^, ^15^, ^58^] Signals of low intensity, in the 5.5 to 6.5 ppm region, corresponding to the vinylic hydrogens of the unreacted MMA_2_, are observed, as expected (77 % conversion after 3 h as shown by GC, Table 2). Together with the signals at 3.5 ppm corresponding to the methoxy hydrogens in both MMA_2_ and the polymer, these assignments confirm a reaction between propagating PE macroradicals and MMA_2_. Contrary to what might have been expected the absence of vinyl protons other than those of residual MMA_2_ indicates that the fragmentation path of Scheme 1 is slow relative to these other processes. However, it does not permit us to discriminate between block and graft architectures in the product although the molar mass evolution suggests that block‐like structure is preferred.

The degree of polymerisation for the polyethylene segment (DP_PE_) as measured by both NMR and SEC was compared. As the absence of well‐resolved end group signal prevents a direct estimation of the number‐average molar mass (*M*_n_) by ^1^H NMR, it was first assumed that the product contains a single methacrylic oligomer unit per chain (200 g mol^−1^). The integral of the signal corresponding to CH_3_−O‐ at ca. 3.5 ppm (I_1_) was thus fixed at a value of 6 H. The integral for the CH_2_ of the PE main chain (I_2_) was then determined to correspond to 275 H. The relative PE molar mass was calculated (Table 2) while taking into account the contribution of unreacted MMA_2_ (Figure 1) to the CH_3_−O‐ signal. The molar masses measured by high‐temperature SEC and NMR gave consistent results (e.g., run 4, *M*_n_(SEC)=2850 vs. *M*_n_(NMR)=2650 g mol^−1^). These results are consistent with the copolymers having one MMA_2_ per chain. Although the presence of one MMA_2_ per chain is a strong indication that a block copolymer structure is formed, it was not yet certain whether graft, block or a mixture of copolymers were formed.

Additional characterisation was employed to further investigate the structure of the product. Although diffusion‐ordered spectroscopy (DOSY) NMR was considered as an analytical method to probe the structure,^[59]^ the high temperature required to solubilise the product is highly challenging for the hardware for DOSY analysis, and to the best of our knowledge, there are no published examples of polyolefin DOSY analysis.^[7]^ The formation of copolymers was confirmed by comparing ^1^H NMR spectra of a mixture of PE/MMA_2_ and of the obtained product in run 9 (Table 2) after precipitation in methanol or acetone. After precipitation, the ^1^H NMR signal at 3.5 ppm, characteristic of the methoxy groups of MMA units, is still present in the formed product whereas in the case of a mixture of MMA_2_ and PE this signal is not visible.

**Scheme 2:**
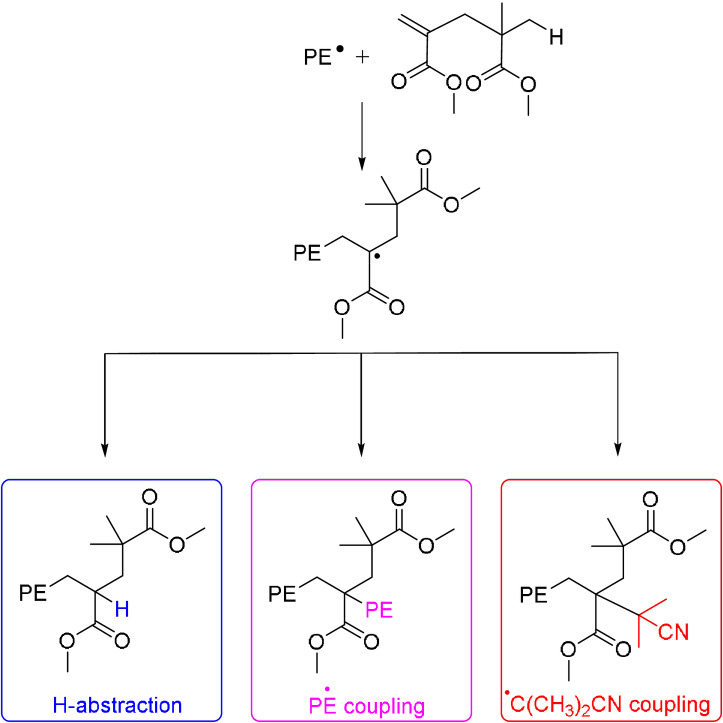
Possible copolymer end‐chains after different termination reactions following the addition of a growing polyethylene chain into MMA_2_.

A series of ^1^H–^1^H and ^1^H–^13^C correlation spectra was carried out to detect and assign key signals consistent with a block copolymer structure (Figure S4–S7). Indeed, the sharp signal at 1.95 ppm assigned to H_a′_ does not seem to correlate to any other hydrogen in the product (Figure S4). In the case of a block copolymer where the intermediate radical is abstracting a hydrogen atom (R=H in Scheme 1), H_a′_ should correlate with H_e′_ in a homonuclear correlation spectroscopy (COSY) analysis. The absence of correlation and the presence of this singlet would be consistent with a graft‐like structure. Figure S5 shows a comparison of ^13^C NMR spectrum of MMA_2_ (Figure S5a) and the polymer obtained with (Figure S5b, for the complete assignments proposed please refer to previous papers)[^11^, ^14^, ^15^] and without MMA_2_ (Figure S5c). The branching notation used, *x*B_y_, was from Galland et al.,^[60]^ in which *x* refers to the number of carbon atoms between the branching and the corresponding carbon and *y* to the number of carbon atoms in the branching. Several signals in Figure S5b that were not present in the homo‐polyethylene (Figure S5c) are observed. Analysis of the 2D NMR ^1^H–^13^C HSQC (Figure S6) and HMBC (Figure S7) spectra allowed for the assignments of these signals to the different carbons of a MMA_2_ bound to a PE chain. The DEPT135 spectrum (Figure S5d) is consistent with the presence of two quaternary carbons (C_u_ and C_y_) and the absence of a CH, which would correspond to a graft architecture. However, this could also be a block copolymer for which the corresponding intermediate radical (Scheme 2) did not abstract an H but terminated by coupling with another growing chain or with tertiary radicals from the decomposition of the initiator. Indeed, two signals, corresponding to nitriles, are present between 123 and 124 ppm (expansion in Figure S5b). They may correspond to two different isobutyronitrile groups from the initiator, one initiating PE chain and the other coupling with the intermediate radicals in Scheme 2. Signals of low intensity between 2.1 and 2.7 ppm (expansion in Figure 1), according to Figure S6 and S7, correlate to CH carbons between 42 and 44 ppm. This could be an indication of the presence of a structure resulting from H‐abstraction, Scheme 2, present in a very small amount.

To gain further insight in the kinetics of the reaction pathway, a computational mechanistic investigation was performed at the MPWB1K DFT level accordingly to previously reported benchmarks.[^61^, ^62^, ^63^] (see SI for the detailed computational procedure). At this level, the Gibbs energy barrier for the addition a PE macroradicals modelled by *n*‐Bu^.^ to ethylene is 2.6 kcal mol^−1^ higher than to the oligomer MMA_2_ (Scheme S1). The formation of the resulting functionalised radical is peculiarly stable (Δ_r_ *G*°=−20.5 kcal mol^−1^) and does not support any reversible character for this addition. Starting from this radical, several reaction pathways were investigated: the energy barrier for the fragmentation into functionalised PE and MMA‐radical is computed to 23.5 kcal mol^−1^, this step is kinetically less favourable by 1.8 kcal mol^−1^ than addition of ethylene. As a reference for accessible energy barriers under the specific experimental conditions, the transition state for the addition of the radical resulting from AIBN fragmentation and MMA_2_ has been optimised. This reaction—that does not occur experimentally—requires to overcome a computed Gibbs energy barrier of 20.5 kcal mol^−1^ to proceed. Thereof, both propagation of a PE chain from the intermediate radical resulting from the addition of PE macroradical onto MMA_2_ and its fragmentation are, respectively, kinetically limited and prohibited. The PE macroradical then most likely evolves via radicals recombination that is computed thermodynamically highly favoured (Δ_r_ *G*°<−40 kcal mol^−1^) and diffusion limited with *k*>10^8^ L mol^−1^ s^−1^, i.e., Δ_r_G^≠^<2 kcal mol^−1^.^[64]^

To conclude, different analytical methods supported that the polymerisation of ethylene in the presence of MMA_2_ leads to the formation of copolymers. It is highly challenging to discriminate between grafts and block structures by analytical methods. However, the comparison of molar mass measured by NMR and SEC indicates that there is only one MMA_2_ per PE chain, corresponding to a block architecture, which is in agreement with DFT calculations. In‐depth ^1^H and ^13^C NMR analyses of chain end confirm that block copolymer structure forms mainly as a result of coupling reaction either with another growing PE chain or with primary radical from AIBN, though the kinetic barriers for these radical couplings could not be properly determined.

The study was extended to the use of higher DP methacrylic oligomers with the aim of forming AB block copolymers based on a PE segment and a PMMA block.

### Copolymerisation of Different Methacrylic Oligomers with Ethylene

We investigated the influence of the methacrylic oligomer molar mass by using MMA_11_ (*M*_n_=1100 g mol^−1^) and MMA_35_ (*M*_n_=3500 g mol^−1^). A statistical copolymer containing on average two MAA units per chain, MMA_12_MAA_2_ (*M*_n_=1400 g mol^−1^), was also employed. Other reaction parameters were kept identical including the replenishing of ethylene throughout the polymerisation to those previously described for MMA_2_. MMA_11_, MMA_35_ and MMA_12_MAA_2_ conversions were determined by ^1^H NMR. For the three studied oligomers, the kinetics are relatively similar (Figure S8 and Table 3). By comparing the conversion of the oligomers and product yields, it was observed that the experiments conducted with the three oligomers followed the same trend (Figure S8c) indicating that they all obey similar reactivity.

**Table 3:** Radical copolymerisation of ethylene in the presence of different ω‐unsaturated methacrylic oligomers.

Run	Methacrylic oligo.	*t* [h]	Methacrylic oligo. conv.^[a]^ [%]	Ethylene cons.^[b]^ [g]	*M*_n_ (NMR)^[c]^ [g mol^−1^]	DP_PE_^[c]^	*M*_n_ (SEC)^[d]^ [g mol^−1^]	*Đ*
1	MMA_11_	0.8	35	0.14	3500	84	2600	7.6
2	MMA_11_	1.5	60	0.29	3550	87	3200	6.2
3	MMA_11_	3.0	85	0.45	3300	76	4400	5.6
4	MMA_11_	4.5	97	1.05	4600	128	5800	4.5
5	MMA_11_	6.0	100	1.80	7300	221	8800	3.7
6	MMA_35_	0.4	12	0.05	4800	46	4900	2.8
7	MMA_35_	0.8	27	0.12	4590	51	4500	2.8
8	MMA_35_	1.5	61	0.33	5050	55	4900	2.8
9	MMA_35_	3.5	97	1.04	10 500	246	10 700	3.9
10	MMA_35_	6.0	100	1.95	11 800	297	12 300	3.7
11	MMA_12_MAA_2_	0.4	20	0.02	2600	43	1150^[e]^	2.8
12	MMA_12_MAA_2_	0.8	38	0.08	2450	36	1700^[e]^	2.2
13	MMA_12_MAA_2_	1.5	60	0.23	3150	62	2200^[e]^	2.7
14	MMA_12_MAA_2_	3.0	90	0.68	3500	75	3850^[e]^	2.5

[a] Calculated by ^1^H NMR. [b] Ethylene consumption=(mass of dried product)−(mass of AIBN)−(mass of methacrylic oligomer). [c] Calculated by assuming that there is one methacrylic oligomer per PE chain, DP_PE_ is the degree of polymerisation and is calculated according to the equation given in Figure 1. [d] Measured using HT‐SEC based on a universal calibration with polystyrene standards. [e] Measured by HT‐SEC using a conventional PE calibration.

The copolymer products were analysed by HT‐SEC (Table 3). Molar masses were measured using a universal calibration method (note that when the molar masses are too low (<5000 g mol^−1^) an accurate measurement is difficult. For copolymers having a MMA_12_MAA_2_ moiety, a conventional PE calibration was used due to solubility issue of the methacrylic oligomer in TCB. Molar masses increased slightly over reaction time, Figure S9, but polymer chains remained short for oligomer conversion below 80 %.

At longer polymerisation times, when almost all of the oligomers have been consumed and the probability of a reaction between an oligomer and a growing chain is low, molar masses tend toward the ones obtained in absence of oligomers (Figure S9d–f) and homopolyethylene is formed. At short polymerisation times, dispersities are high and are broadened by the presence of unreacted oligomers.

^1^H and ^13^C NMR spectroscopy were also performed. Assuming there is one oligomer unit per chain, the molar masses calculated by NMR correspond well to those determined by SEC (Table 3). As it was the case for MMA_2_, this is an indication that the obtained structure is a block copolymer. At short polymerisation times, solubility issues with unreacted MMA_12_MAA_2_ and the utilisation of a PE calibration make it difficult to determine the molar masses of the copolymer by SEC, and may explain why there are outliers for some experiments (Table 3). However, the presence of higher DP methacrylic oligomers results in the NMR spectrum analysis being more difficult. The signal at 1.95 ppm is no longer visible, hidden by the CH_2_ signal from the oligomer (e.g. MMA_11_ in Figure S10). Too many signals were present to conduct thorough ^13^C NMR analysis, but a comparison with the spectrum recorded when MMA_2_ is used shows similar characteristic signals (Figure S11 and S12).

An observed apparent chain transfer constant *C*_s′_ was calculated using ethylene and methacrylic oligomer consumptions (Table S2 and Figure S13).[^15^, ^65^, ^66^] *C*_s′_ is the ratio between the rate transfer of a polyethylenic propagating chain to the methacrylic oligomer and the propagating rate of ethylene. *C*_s′_ values of 55.6, 56.4, 52.8 and 56.6 were calculated for MMA_2_, MMA_11_, MMA_35_ and MMA_12_MAA_2_, respectively. Considering the error margin, *C*_s′_ are identical for all oligomers with an average value=55.4 that is in good agreement with the value of 45 obtained considering computed energy barriers (Scheme S1) corrected by reaction symmetry numbers.^[67]^ As a consequence, it can be assumed that there is almost no chain length dependence. Previously, Moad and Rizzardo have demonstrated that MMA_2_ had a substantially lower chain transfer constant than the trimer or oligomers of higher molar mass.^[68]^ These high values obtained here indicate that transfer to oligomers is favoured compared to propagation under these conditions, and leads to a PE chain with molar mass increasing upon consumption of the oligomer as seen with MMA_2_, in agreement with the formation of block copolymers.

### Copolymerisation of Ethylene with Different Concentrations of MMA_11_

Subsequently, we studied the influence of the initial MMA_11_ concentration in the reaction on the final copolymer properties. Polymerisations were carried out with a molar ratio of [MMA_11_]/[AIBN]/[ethylene]=3/1/1900 in place of 1/1/1900 previously. All other parameters were kept constant. A higher MMA_11_ concentration leads to a sharp fall in yield (after 6 hours, 1.80 vs. 0.50 g for [MMA_11_]/[AIBN] ratios of 1 and 3, respectively), Figure S14 and Table 4. While this behaviour may not be consistent with the MMA_11_ acting as conventional chain transfer, it shows that the transfer is degradative and not able to reinitiate chains. This is consistent with the synthetic scheme provided in Scheme 2 for which, after the first addition, the intermediate radical terminates without further propagation. For both concentrations, MMA_11_ conversion versus ethylene consumption trend is similar (Figure S14c) and close *C*_s′_ values were calculated (56.4 vs. 60.6 at [MMA_11_]/[AIBN] ratios of 1 and 3, respectively). These data further support the hypothesis that only the ω‐vinyl functional oligomer end‐chain and the type of comonomer used influence its reactivity.[^48^, ^69^]

**Table 4:** Radical copolymerisation of ethylene with different molar ratios of [MMA_11_]/[AIBN] of 3.

Run	*t* [h]	MMA_11_ conv.^[a]^ [%]	Ethylene cons.^[b]^ [g]	*M*_n_ (NMR)^[c]^ [g mol^−1^]	DP_PE_^[c]^	*M*_n_ (SEC)^[d]^ [g mol^−1^]	*Đ*
1	0.8	8	0.00	2000	31	450	3.1
2	1.5	24	0.02	2100	33	550	2.2
3	3.0	49	0.18	2050	35	1300	2.5
4	4.5	72	0.39	2150	38	1200	4.2
5	6.0	86	0.50	2400	44	2300	5.0
6	15.0	100	2.63	4750	132	5800	3.8

[a] Calculated by ^1^H NMR. [b] Ethylene consumption=(mass of dried product)−(mass of AIBN)−(mass of MMA_11_). [c] Calculated by assuming that there is one MMA_11_ per PE chain, DP_PE_ is the degree of polymerisation and is calculated according to the equation given in Figure 1. [d] Measured by HT‐SEC using a conventional PE calibration.

The evolution of the molar mass distribution presents a similar behaviour to that at the lower MMA_11_ concentration (Figure S15). The MMA_11_ is consumed over the course of the polymerisation, leading to an increase of molar masses and the formation of homopolyethylene when all of the MMA_11_ has been consumed. As a result of a high MMA_11_ concentration favouring the transfer reaction, there is a sharp decrease of molar mass at a short polymerisation time (Table 3 and 4).

^1^H and ^13^C NMR analyses were similar to those obtained with [MMA_11_]/[AIBN] ratios of 1. Only the signal at 123.5 ppm is more intense than previously (Figure S12b,d). Shorter polyethylene chains can increase the flexibility at the polar/apolar interface and thus lead to a better relaxation of the carbon of the nitrile.

### Thermal Analysis

**Figure 2:**
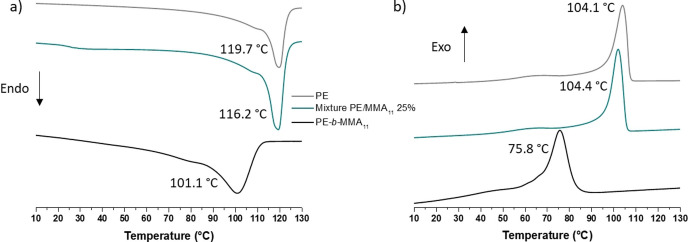
DSC a) cooling and b) heating. Comparisons of a PE (run 4 in Table 2), blend of PE and MMA_11_ (75/25 w/w) and copolymer of PE and MMA_11_ (run 5 in Table 4).

PE homopolymer and copolymers (run 5 in Table 4) together with physically mixed samples of MMA_11_ and PE (run 4 in Table 2) were analysed by DSC (Figure 2). As expected, the melting and crystallisation temperatures of PE (*T*_m_=119.7 and *T*_c_=104.1 °C, respectively) are similar in the MMA_11_/PE blend (*T*_m_=116.2 and *T*_c_=104.4 °C). The glass transition temperature of MMA_11_ (*T*_g_=40.3 °C) could not be determined in the MMA_11_/PE mixture as it is overlapping with the PE melting temperature. However, significant changes are observed in the case of the block copolymers. More specifically, the melting and crystallisation temperatures of the block copolymers (*T*_m_=101.1 and *T*_c_=75.8 °C) decrease between 15 °C and 25 °C compared to the PE homopolymer. Such differences can also be seen for the other block copolymer samples (Figure S16), for which the melting temperature increases with polymerisation time. This correlates with the increase of molar masses of PE segment with the polymerisation time (Figure S17). Results were compared with melting temperatures of well‐defined homopolyethylenes published by Pak and Wunderlich.^[70]^ The gap between both curves (Figure S17) can be explained by the branched nature of PE segments compared to the linear PE studied by Pak and Wunderlich. This agreement between these values corroborates the assumption than there is only one methacrylic oligomer per PE chain and therefore supports the formation of block copolymers.

### SAXS

SAXS/WAXS analysis of PE‐*b*‐MMA_11_ block copolymer (run 5 in Table 4) was carried out and compared with pure PE (run 4 in Table 2) and a blend of PE and MMA_11_. PE bulk scattering intensities show the presence of a broad peak corresponding to the presence of crystalline domains dispersed in the amorphous phase (Figure S16). The characteristic length of the crystallite measured by SAXS (*ξ*≈55 nm) corresponds to the characteristic domain sizes obtained from the WAXS spectrum using the Scherrer equation (cf. Figure S17 and Table S3). The scattering intensities of the PE/MMA_11_ blend (Figure S18) show more complex features: a peak around 0.03 Å^−1^ (*ξ*≈80 nm and *d*≈200 nm) characteristic of the dispersed PE phases, followed at higher q‐range by two wavelets characteristic of a cylindrical form factor (*R*=56 nm and *L*=35 nm), most likely the PE crystallites. Once again, the characteristic domain sizes (*R*≈40 nm and *L*≈20 nm), determined using the Scherrer equation from the WAXS spectra (Figure S19 and Table S4), confirmed the size of the PE crystallites observed by SAXS. All these results are consistent with the presence of semi‐crystalline PE nodules embedded within a MMA_11_ matrix. Finally, the scattering intensities of the block copolymer of PE‐*b*‐MMA_11_ (Figure S20) were fitted by a Guinier form factor (i.e. spheroid object, with a radius of gyration, *R*_g_≈30 nm). The PE domains are smaller compared to the PE/MMA_11_ blend and more dispersed (no correlation peak characteristic of the distance between domains). At higher *q*‐range, a *q* ^−2^ slope is observed corresponding to planar objects which probably correspond to PE crystalline lamella. However, the precise form factor is not clearly visible because the sample appears to be less crystalline than previous samples. Indeed, the WAXS spectrum (Figure S21 and Table S4) shows only 3 crystalline peaks, and the crystallinity index was found to be around 17 % (compared to around 50 % for bulk PE and the PE/MMA_11_ blend). These results are in good agreement with crystallinities measured by DSC (12 and 47 % for PE‐*b*‐MMA_11_ block copolymer and bulk PE, respectively). Furthermore, the thickness of the PE lamella for PE‐*b*‐MMA_11_ is around 25 nm which corresponds to a PE segment with ca. 100 monomers (i.e. *M*_n_≈2800 g mol^−1^) which is in good agreement with the molar mass of the PE segments measured by SEC and NMR. The overall nanostructure is in good agreement with a block copolymer structure where the MMA_11_ segment hinders the crystallisation of the PE segment. Therefore, PE‐*b*‐MMA_11_ probably behaves as a polymeric surfactant, reducing surface tension between MMA_11_ and PE phases, leading to a more dispersed PE phase and inducing PE surpercooling crystallisation.^[71]^

## Conclusion

In this study, the synthesis of copolymers based on methacrylates and ethylene using a combination of CCTP and FRP has been investigated. MMA_*n*_ produced by CCTP is constantly consumed in the FRP of ethylene. The intermediate radical formed by addition of the polyethylenyl propagating radical onto MMA_*n*_ was shown to terminate by H‐abstraction rather than propagate leading to a MMA_*n*_‐*b*‐PE block copolymer. This conclusion is the result of an accurate characterisation of the obtained products through several NMR and HT‐SEC analyses and is confirmed by calculations performed at the DFT level. A broad range of block copolymers with various PE molar mass segments can easily be obtained using MMA_*n*_ with different compositions and concentrations. This allows to isolate block copolymers using established radical polymerisation techniques and combining properties of both blocks as shown by DSC and SAXS/WAXS studies and unattainable by conventional copolymerisation of the MMA and ethylene.

## Conflict of interest

The authors declare no conflict of interest.

## Supporting information

As a service to our authors and readers, this journal provides supporting information supplied by the authors. Such materials are peer reviewed and may be re‐organized for online delivery, but are not copy‐edited or typeset. Technical support issues arising from supporting information (other than missing files) should be addressed to the authors.

Supporting Information
