# Supplementary material for: Block Copolymers Based on Ethylene and Methacrylates Using a Combination of Catalytic Chain Transfer Polymerisation (CCTP) and Radical Polymerisation
Source: Angew Chem Int Ed Engl. 2021 Oct 22;60(48):25356–64. doi: 10.1002/anie.202108996 (PMC9298203; doi:10.1002/anie.202108996)
Supplement: Supplementary file 1 — Supporting Information [file ANIE-60-25356-s001.pdf]

Angewandte Chemie
Eine Zeitschrift der Gesellschaft Deutscher Chemiker

Supporting Information

Block Copolymers Based on Ethylene and Methacrylates Using a Combination of Catalytic Chain Transfer Polymerisation (CCTP) and Radical Polymerisation

Florian Baffie[+], Georgios Patias[+], Ataulla Shegiwal, Fabrice Brunel, Vincent Monteil, Ludmilla Verrieux, Lionel Perrin, David M. Haddleton, and Franck D'Agosto**

anie_202108996_sm_miscellaneous_information.pdf

Author Contributions

F.B. and G.P performed the experiments and carried out the characterisations. L.P. and L.V. performed the DFT studies. F.B. analysed the data from SAXS/WAXS. All authors discussed the results and commented on the manuscript. F.D. and D.M.H supervised and designed the research. F.B., G.P., F.D. and D.M.H. wrote the manuscript with suggestions from all the authors.

Supporting Information

Table of content

Experimental section 1
Copolymerisation of methacrylic dimer (MMA_2) with ethylene 5
Determination of structure formed during the ethylene copolymerization with MMA_2 via NMR studies 6
Molecular modelling 9
Copolymerisation of different methacrylic functional oligomers with ethylene 23
Calculation of the chain transfer constant C_s' (= k_{tr}/k_p) for methacrylic oligomers [12–14] 26
Copolymerisation of ethylene with different concentrations of MMA_{11} 28
DSC of the different copolymers synthesized 29
SAXS/WAXS analysis of the different copolymers 31
References 39

Experimental section

Materials.

2,2'-Azobis(isobutyronitrile) (AIBN, Aldrich, 98 %) and dodecane (Aldrich, 99 %) were used as received. Dimethyl carbonate (DMC, Aldrich, 99 %) was used after bubbling with argon for at least 12 h. Methyl methacrylate (MMA, Aldrich, ≥ 99%), initiator V-601 (dimethyl-2,2'-azobis(2-methyl propionate), Wako Chemicals) were used as received. 4,4'-azobis(4-cyanovaleric acid) (Aldrich, ≥ 98%) and sodium dodecyl sulfate (MP Biomedicals, LLC) were used as received. The catalyst bis[(difluoroboryl)dimethyl glyoximato]cobalt(II) (CoBF) was synthesized as previously described.[1]

Methods

All NMR experiments were performed at 90 °C in a mixture of tetrachloroethylene and deuterated benzene (2:1 v:v) or at 25 °C in $CDCl_3$ using a Bruker Avance 400 spectrometer (^1H: 400.13 MHz; ^{13}C: 100.61 MHz). The temperature was calibrated with a tube containing 80% ethylene glycol and 20% DMSO-d6. Proton NMR spectra were recorded with a 5 mm BBFO+ probe with a z-gradient coil. Carbon NMR spectra were recorded with a 10 mm SEX probe, ^{13}C selective with a z-gradient coil. Chemical shifts are given in parts per million (ppm) with the deuterated solvent used as internal standard.

Molar mass measurements of PE were performed using a Viscotek High-Temperature Triple Detection SEC (HT-SEC) system (Malvern Instruments) that incorporates a differential refractive index, a viscometer, and a light scattering detector. 1,2,4-Trichlorobenzene (TCB) was used as the mobile phase at a flow rate of 1 mL min^{-1}. TCB was stabilized with 2,6-di(tert-butyl)-4-methylphenol. The separation was carried out on three mixed bed columns (PLgel Olexis 300 × 7.8 mm from Malvern Instrument) and a guard column (75 × 7.5 mm). Columns and detectors were maintained at 150 °C. Sample volumes

of 200 µL were analyzed at concentrations of 3 mg mL^{-1}. The Omnisec software was used for data acquisition and data analysis. The molar mass distributions (MMD) were calculated by means of a conventional calibration curve on the basis of linear polyethylene standards from 300 to 130 000 g mol^{-1} (Polymer Standards Service).

Gas chromatography (GC) analyses were conducted on a GC HP instruments 6890 equipped with a flame ionization detector (FID) and an Agilent 19091J-413 HP-5 column (30 m × 320 µm × 0.25 µm). Injector and detector temperatures were set to 250 °C. The initial and final column temperatures were respectively 40 °C and 250 °C with a heating rate of 20 °C min^{-1} and a final isotherm of 2 min at 250 °C. The internal standards used was dodecane.

DSC analyses were performed on a Mettler Toledo DSC 3. Measurements were carried out by two successive heating and cooling cycles (5 K min^{-1} for the first cycle) with temperatures ranging from 10 to 220 °C. Crystallization, melting and glass transition temperatures were recorded on the second cycle.

Small-angle X-ray scattering (SAXS) measurements were made using a Xenocs Xeuss 2.0 equipped with a micro-focus Cu Kα source collimated with Scatterless slits. The scattering was measured using a Pilatus 300k detector with a pixel size of 0.172 mm x 0.172 mm. The wide angle x-ray scattering (WAXS) data was collected using a Pilatus 100k mounted at an angle of 36 º from the beam and a distance of 162(2) mm from the sample. The distance between the detectors and the sample was calibrated using silver behenate ($AgC_{22}H_{43}O_2$). The magnitude of the scattering vector (q) is given by $q = (4\pi \sin(\theta))/\lambda$, where 2θ is the angle between the incident and scattered X-rays and λ is the wavelength of the incident X-rays. This gave a q range for the SAXS detector of 0.005 $Å^{-1}$ and 0.16 $Å^{-1}$. A radial integration of the 2D scattering profile was performed using FOXTROT software and the resulting data corrected for the absorption, sample thickness and background. When required, samples were mounted between sticky Kapton windows.

Geometry optimization of reactants and transition states were carried out without any symmetry restrictions at the DFT level of theory using the global hybrid meta-GGA functional MPWB1K.[2,3] For each compound, a conformational sampling has been performed by hand. Only the most stable conformers are reported. All atoms were represented by all electron polarized def2-TZVP basis sets.[4] Solvation by DMC was implicitly represented during optimization using dibutylether as a model (similar ε_r) and for which parameters are available for the SMD method.[5] According to previous benchmark studies no dispersion correction was added.[6–8] Analytical frequency calculations were carried out to verify the nature of the extrema (minimum or transition state). Gibbs energies have been computed within the harmonic approximation and estimated at 298.15 K and 1 atm. Standard state conversion has been applied and evaluated at 343K.[9] Additionally, energy barriers have been corrected by reaction symmetry numbers in order to account for the statistic degeneracy of transitions states.[10] Criteria for SCF convergence, geometry optimization, and integration grids are set to default values. All these computations have been performed with the gaussian09 suite of programs.[11]

General procedure for the preparation of poly(methyl methacrylate) ω-functional oligomers (MMA_{n},) via catalytic chain transfer polymerisation (CCTP)

Four different ω-functional oligomers were prepared either by solution or emulsion polymerization according to described protocols.[12,13] For a CCTP carried out in solution, bis[(difluoroboryl)dimethyl glyoximato]cobalt(II) (CoBF, 0.09 mg, 5 ppm relative to monomer) or bis[(difluoroboryl)dimethyl phenyl-glyoximato]cobalt(II) and a stirring bar were charged into a 100 mL round bottom flask. The flask was purged with nitrogen for one minute. Subsequently, 10 mL of methyl methacrylate (MMA), previously deoxygenated for 30 minutes by bubbling with nitrogen, were added to the flask *via* a deoxygenated syringe. The mixture was stirred under a nitrogen atmosphere until dissolution of the catalyst. Meanwhile, dimethyl-2,2'-azobis(2-methyl propionate) (V601, 107 mg, 1 mol% relative to monomer) was dissolved in 10 mL toluene (1/1 v/v to monomer) and this solution charged into a 50 mL round bottom flask and bubbled with nitrogen for 30 min. Subsequently, the 100 mL flask with the monomer and catalyst was heated to 75 °C under an inert nitrogen atmosphere. When the temperature of the catalyst solution reached 75 °C, the initiator solution was added. The reaction was allowed to continue for 6 hours with continuous stirring. MMA dimer (MMA_2) was recovered after distillation whereas poly(methyl methacrylate) with higher molar mass (1100 g mol^{-1}, MMA_{11}) was recovered by precipitation in methanol. MMA_2 after distillation is composed of three products, MMA dimer (97%), trimer (3%), which were analysed with ^{1}H and ^{13}C NMR spectroscopy as well as Gas Chromatography (GC) (Figure S1 and Table S1) as the percentage of each oligomer in the mixture could play a role in the synthesis of copolymers.

Table S1. MMA_2, synthesised by CCTP, composition before and after distillation, determined by GC

Monomer	Product Mixture	Distilled MMA dimer	Elution time (min)
MMA dimer	65 %	97 %	4.4
MMA trimer	30 %	1.4 % (internal double bond) 0.1 % (initiator terminated) 1.5 % (*ω double bond*)	6.5 / 6.90 / 7.02
MMA tetramer	5 %	0 %	9.26

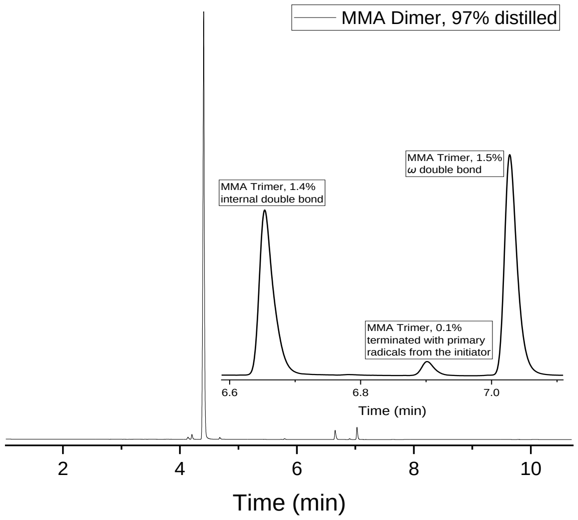

Figure S1. GC-FID of MMA_2 recovered by vacuum distillation.

For a CCTP in emulsion, 22.4 mg of CoBF and a stirring bar were charged into a 100 mL round bottom flask. The flask was purged with nitrogen for at least one minute. Subsequently, MMA (96.3 mL) and optionally MAA (10.7 mL) when copolymers were targeted, previously deoxygenated for at least 30 min, were added to the flask *via* a deoxygenated syringe. The mixture was stirred under a nitrogen atmosphere until the total dissolution of the solids. Meanwhile, 4,4'-azobis(4-cyanovaleric acid) (ACVA, 1.35 g), sodium dodecyl sulfate (SDS, 2.1 g) and 250 mL of deionized water were charged into a three-neck, 500 mL double jacketed reactor, equipped with a thermometer and an overhead stirrer. The mixture was bubbled with nitrogen for at least 30 min. Subsequently, the mixture was heated under inert atmosphere. When the temperature of the mixture reached 75 °C, the addition of the solution of monomer and cobalt complex is performed with a syringe pump (feeding rate = 1.866 mL min^{-1}, feeding time = 60 min). After the end of the addition, stirring continued for another 60 min under the same temperature and stirring rate. The final products (MMA_{35} and $MMA_{12}MAA_2$) were recovered by lyophilisation using the freeze-dryer.

The number average molar mass (M_n) of the ω-functional oligomers was calculated from ^1H NMR by integrating the vinyl resonances (6.17 and 5.44 ppm) against the methoxy signal (3.57 ppm). ^1H NMR (400 MHz, $CDCl_3$): δ (ppm) = 6.17 (s, 1H), 5.44 (s, 1H), 3.57 (s, 3H, broad), 2.00–1.80 (s, 2H, br), 1.20 (s, 3H, mm), 1.01 (s, 3H, mr), 0.82 (s, 3H, rr). The dispersity and molar masses of the final products were determined by SEC.

General procedure for the polymerisation of ethylene

The reaction was conducted in a stainless-steel reactor (160 mL, from Parr Instrument Co.) equipped with a thermometer, a pressure sensor, a mechanical stirring apparatus, and safety valves. In a typical polymerisation procedure, AIBN (50 mg, 0.30 mmol) was dissolved in degassed DMC (50 mL). The solution was later introduced into an injecting chamber. The chamber was then pressurised at 30 bar of ethylene, and open into a preheated autoclave at 70 °C. Ethylene gas was then introduced to the reactor until the desired pressure of 80 bar was reached. The reaction medium was stirred at 600 rpm. In order to manage the polymerisation safely over 50 bar of ethylene, we use a 1.5 L intermediate tank. The tank is cooled at -20°C during a filling step with the ethylene bottle. After isolation, it is then heated back and connected to the reactor to get the desired pressure. This tank was used to charge the reactor and to maintain a constant pressure of ethylene in the reactor by successive manual ethylene additions. After the desired time, the stirring was slowed down, and the autoclave was cooled with iced water. When the temperature inside the autoclave dropped below 25 °C, the remaining pressure was carefully released. The contents of the reactor were collected with acetone or toluene. The evaporation of the solvent gave the polymeric product

General procedure for the copolymerisation of methacrylic oligomers with ethylene

In a typical polymerisation procedure, a solution of methacrylic oligomers (0.30 mmol) and AIBN (50 mg, 0.30 mmol) in DMC (50 mL) was degassed by bubbling with argon for 30 minutes. The remainder of the procedure was the same as previously described for ethylene polymerisation.

^1H NMR (400 MHz, TCE/C_6D_6): δ (ppm) = 3.20-3.60 (br), 1.40-0.90 (br), 0.90-0.60 (br). The dispersity and molar masses of the final products were determined by HT-SEC.

General procedure for precipitation of the copolymer of MMA_2 and ethylene

The polymer (100 mg) was dissolved in 10 mL of toluene at 90°C and stirred for two hours. The solution was subsequently poured in 100 mL of methanol or acetone, and a white solid precipitated. The solid was filtered and dried under vacuum to afford a white powder. The solid was analyzed by ^1H NMR and HT-SEC.

Copolymerisation of methacrylic dimer (MMA_2) with ethylene

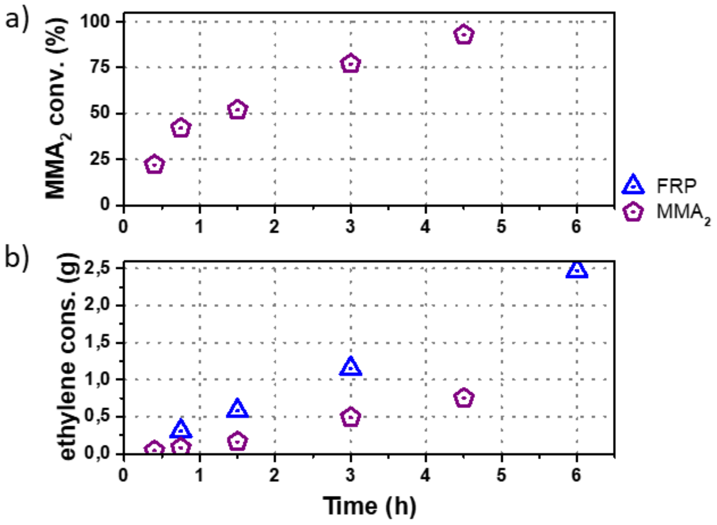

Figure S2. a) MMA_2 conversion as a function of polymerisation time as determined by GC and b) ethylene consumption in the presence and absence of MMA_2. Polymerisation were conducted at 80 bar, 70 °C in DMC using AIBN as initiator. The molar ratio of [MMA_2]/[AIBN]/[ethylene] was set to 1/1/1900.

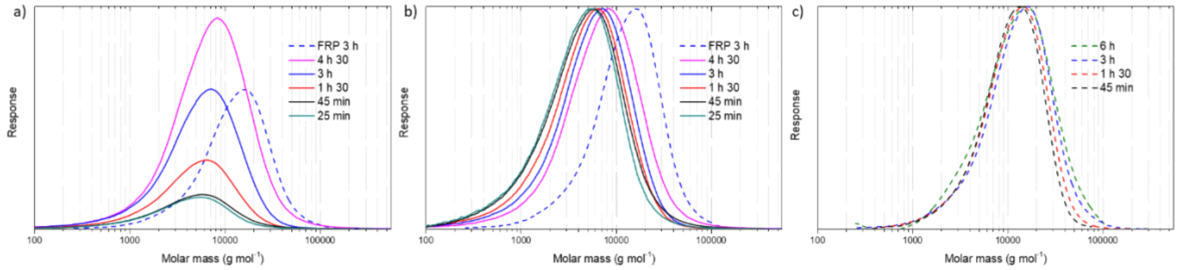

Figure S3. Molar mass distributions calculated with PE calibration of polymer obtained during polymerisation of ethylene in the presence of MMA_2 at 70 °C and 80 bar of ethylene pressure after the indicated polymerisation time (Table 2) a) normalised by yields, b) normalised by area and c) without MMA_2 normalised by area.

Determination of structure formed during the ethylene copolymerization with MMA_2 via NMR studies

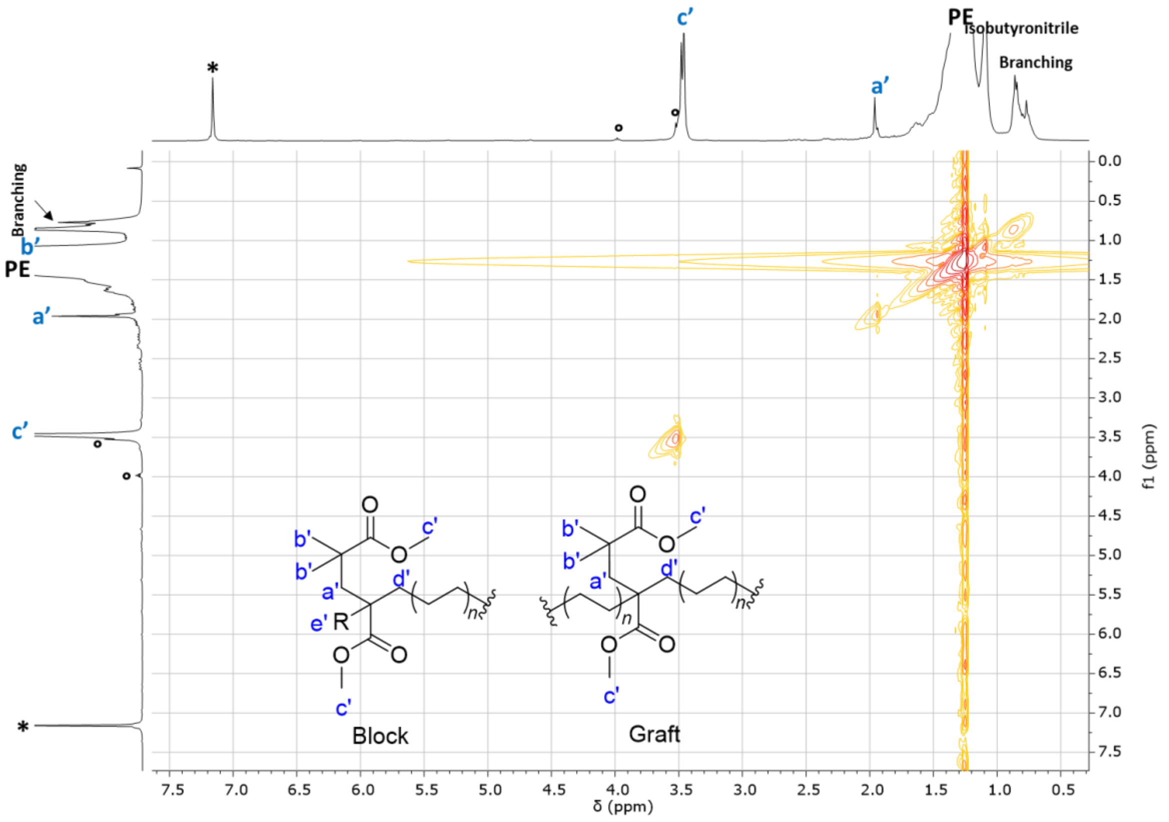

Figure S4. ^1H-^1H COSY NMR from run 8 in Table 2 in TCE/C_6D_6 at 90°C. Isobutyronitrile stems from the chain-ends of the PE initiated from AIBN.

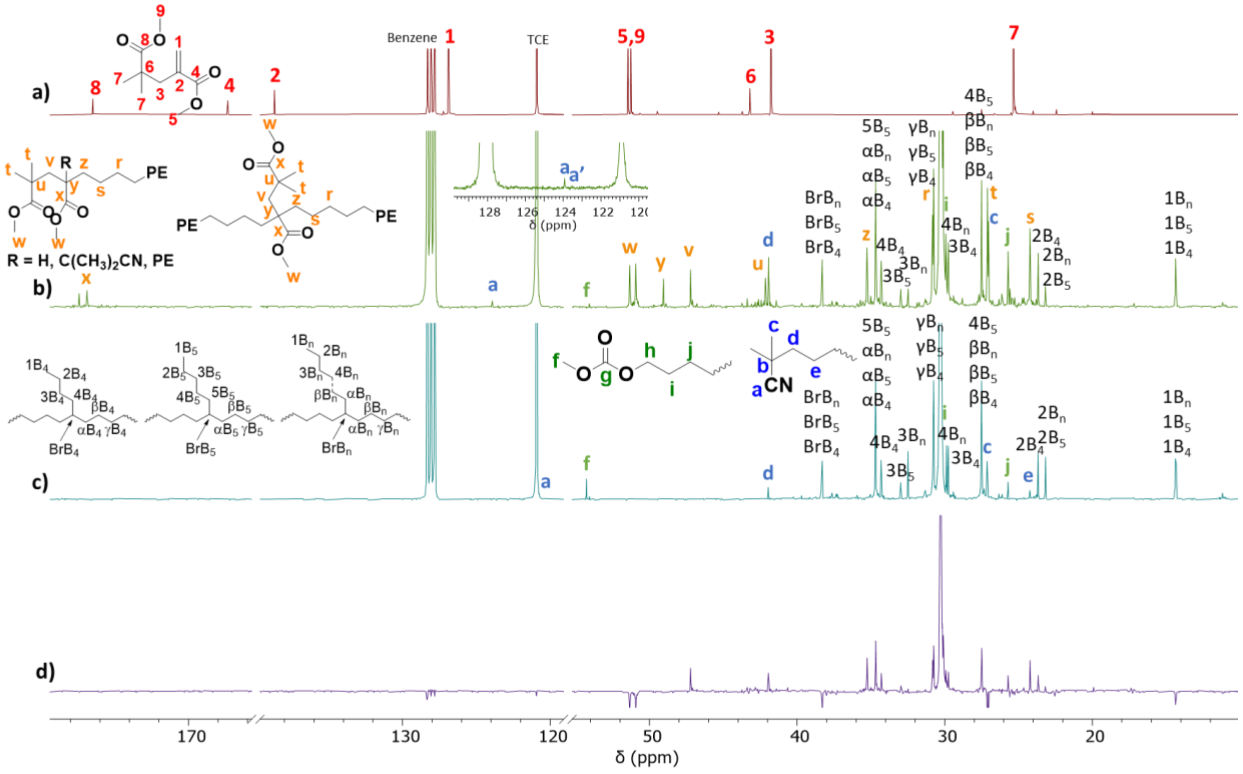

Figure S5. ^{13}C NMR spectra of a) unreacted MMA_2, b) copolymerisation of ethylene with MMA_2 (run 8, Table 2), c) homopolyethylene of run 4, and d) DEPT135 analysis of the polymer recovered in run 4 in TCE/C_6D_6 at 90°C

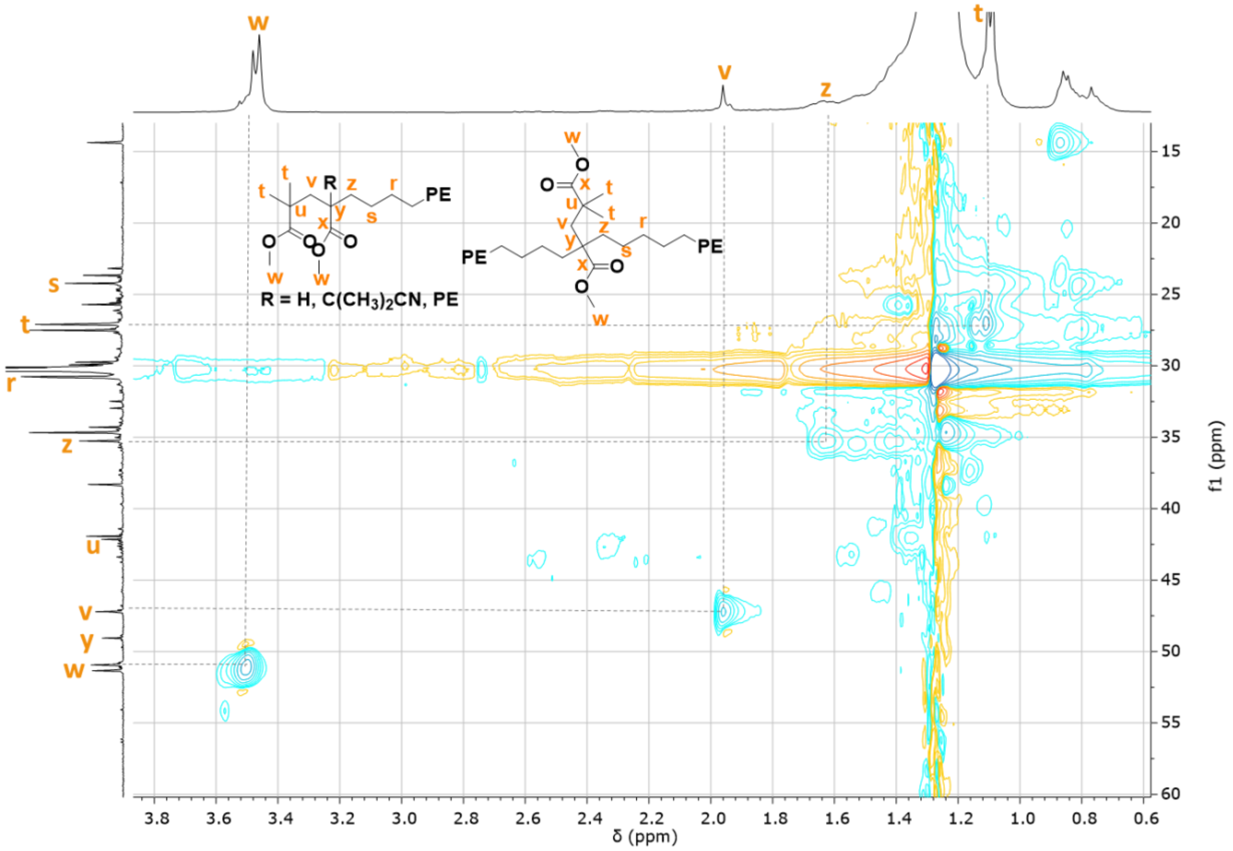

Figure S6. ^{1}H-^{13}C HSQC NMR from run 8 in Table 2 in TCE/C_6D_6 at 90°C.

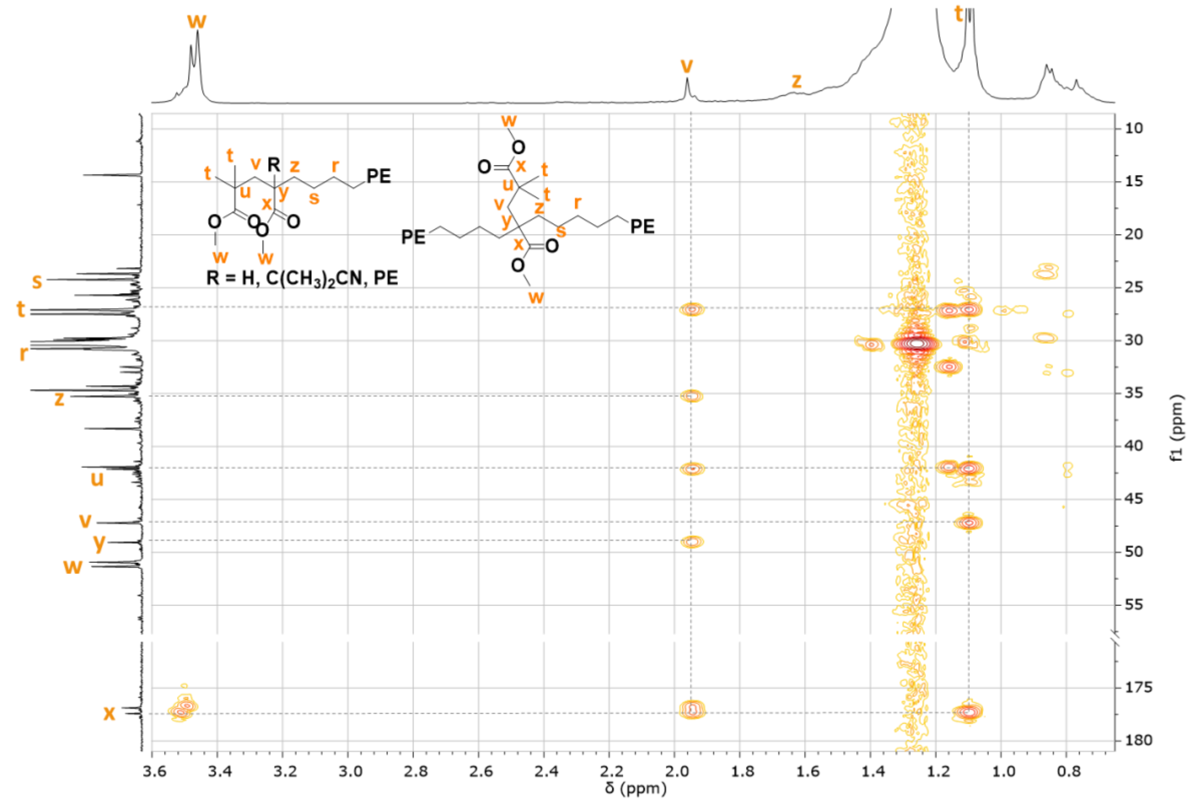

Figure S7. ^{1}H-^{13}C HMBC NMR from run 8 in Table 2 in TCE/C_6D_6 at 90°C.

Molecular modelling

Energy profiles

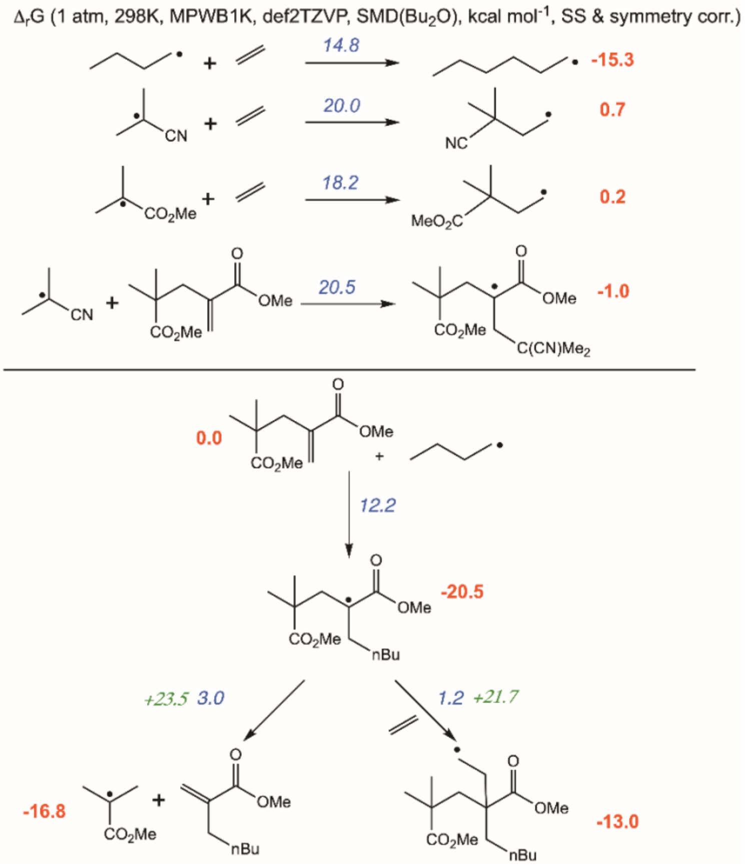

Scheme S1. Gibbs Energy profiles computed at the MPWB1K DFT level. Thermodynamics is in red, kinetics in blue. Gibbs Energy variation are relative to starting reactants. Green values refer to the Gibbs energy barrier of the quoted elementary step.

Cartesian coordinates and energies

Reaction statistic factors σ

$$\sigma = \frac{\sum_i \sigma_{rot,R}^i}{\sigma_{rot,TS}}$$

$\sigma_{rot,R}^i$ rotation symmetry number of reactants; $\sigma_{rot,TS}$ rotation symmetry number of TS

σ = 4 for addition to C_2H_4; σ = 2 for addition to MMA_2 ; σ = 1 otherwise.

Application within the Eyring equation

$$k = \sigma \frac{k_B T}{h} e^{-\frac{\Delta G^{\neq}}{RT}}$$

Standard state conversion

$$\Delta G_{C^{\circ}} = \Delta G_{P^{\circ}} + RTln\left\{\left(\frac{RT}{P}\right)^{\Delta n}\right\}$$

$\Delta G_{C^{\circ}}$and $\Delta G_{P^{\circ}}$are respectively the Gibbs energy the standard state to imply all species at 1 M and 1 atm. Δn is the molecularity of the reaction.

For 80 bar and 343K, and $\Delta n = -1$ (A + B -> C), the standard state conversion correction is of -2.2 kcal mol^{-1}.

C_2H_4

6

E: -78.5631588020 ; H: -78.507053 ; G: -78.532515

C	-1.254713	-0.202747	0.016543
H	-0.741812	-1.150870	0.016543
H	-2.332264	-0.233448	0.016543
C	-0.597420	0.935736	0.016543
H	-1.110321	1.883858	0.016543
H	0.480131	0.966436	0.016543

DMC

12

E: -343.594103008 ; H: -343.488483 ; G: -343.526903

C	-1.097288	-0.173500	-0.063232
O	-0.043102	-0.101046	-0.619540
O	-2.272915	-0.031031	-0.632138
O	-1.272813	-0.410417	1.217143
C	-0.081262	-0.576142	1.962391
H	-0.391853	-0.784773	2.976073
H	0.501012	-1.402762	1.574354
H	0.514111	0.328636	1.935786
C	-2.234382	0.233571	-2.021840
H	-1.742580	-0.573376	-2.551498
H	-3.264758	0.310574	-2.337758
H	-1.713562	1.162752	-2.219181

MMA_2

30

E: -691.527545785 ; H: -691.253369 ; G: -691.313775

C	-3.783934	2.183751	-0.052108
H	-4.760321	1.761515	0.105957
H	-3.713732	3.061935	-0.672640
C	-2.700874	1.641625	0.477286
C	-2.806394	0.390623	1.265086
O	-1.871816	-0.311957	1.522489
O	-4.042279	0.109322	1.642205

C	-4.206945	-1.106279	2.345018
H	-5.258807	-1.172561	2.584230
H	-3.912058	-1.948179	1.729832
H	-3.617283	-1.106994	3.253994
C	-1.329055	2.192189	0.281808
H	-0.622441	1.368376	0.268708
H	-1.279635	2.691202	-0.678536
C	-0.898968	3.194110	1.369959
C	-1.731716	4.463406	1.291408
H	-1.373967	5.200570	2.004114
H	-1.696243	4.901827	0.300271
H	-2.765478	4.242527	1.535241
C	-0.996927	2.595264	2.754444
H	-0.631918	3.294515	3.497842
H	-2.032640	2.369951	2.990739
H	-0.417858	1.683578	2.835619
C	0.541167	3.561427	1.098528
O	1.442124	3.446025	1.874414
O	0.708197	4.052146	-0.120976
C	2.028288	4.429779	-0.456900
H	2.392438	5.194658	0.218693
H	2.693876	3.575616	-0.414719
H	1.983792	4.815115	-1.465764

Me$_2$CCN$^\bullet$

11

E: -210.695053475 ; H: -210.597671 ; G: -210.635132

C	-3.015207	-1.383000	-0.152054
C	-1.768661	-2.020474	0.323373
H	-1.142681	-2.300779	-0.522053
H	-1.197522	-1.373787	0.976035
H	-1.996223	-2.940177	0.860080
C	-3.914918	-2.117474	-1.066875
H	-4.783689	-1.536423	-1.346175
H	-3.381091	-2.401586	-1.972120
H	-4.251030	-3.042366	-0.600827
C	-3.341568	-0.108436	0.262909
N	-3.615214	0.958440	0.609752

Me$_2$CCN$^\bullet$_C_2H_4_TS

17

E: -289.241727253 ; H: -289.087174 ; G: -289.130717

C	-1.412022	-0.074513	0.251156
H	-0.785038	-0.950263	0.243161
H	-2.470152	-0.229621	0.123605
C	-0.901854	1.157405	0.482945
H	-1.511317	2.029916	0.313797
H	0.163000	1.315002	0.431762
C	-0.909630	1.476541	2.616281
C	-2.352127	1.359662	2.971152
H	-2.711381	0.368899	2.714811
H	-2.492311	1.499948	4.041761

H	-2.958456	2.096469	2.457433
C	0.001220	0.410236	3.119661
H	1.001719	0.505144	2.714321
H	0.074643	0.454560	4.205180
H	-0.395059	-0.563492	2.852841
C	-0.380809	2.783153	2.592505
N	0.051574	3.843621	2.520164

$Me_2C(CN)CH_2\underline{C}H_2$

17

E: -289.277255225 ; H: -289.120465 ; G: -289.163007

C	-1.414657	-0.125465	0.352241
H	-0.748909	-0.964836	0.256892
H	-2.462501	-0.293754	0.176839
C	-0.912929	1.200045	0.749528
H	-1.520511	1.982938	0.303335
H	0.106355	1.336652	0.398920
C	-0.912800	1.425738	2.282297
C	-2.325083	1.363438	2.839616
H	-2.726297	0.368747	2.678110
H	-2.329690	1.564650	3.904994
H	-2.973882	2.082068	2.350985
C	-0.020647	0.411175	2.977545
H	0.989553	0.439959	2.584121
H	0.019974	0.598313	4.044611
H	-0.425559	-0.582534	2.819108
C	-0.375372	2.760810	2.520670
N	0.046350	3.806162	2.696521

$Me_2\underline{C}CO_2Me^{\bullet}$

16

E: -346.327602721 ; H: -346.182191 ; G: -346.226321

C	-0.699248	3.357689	1.737406
C	-1.839453	4.179587	1.296468
H	-2.476111	4.433066	2.140401
H	-1.526975	5.087375	0.799477
H	-2.465400	3.619821	0.598953
C	-0.917934	2.298661	2.734854
H	-1.259143	2.724832	3.678019
H	-1.704813	1.619531	2.408351
H	-0.014775	1.732029	2.914865
C	0.622311	3.526120	1.195490
O	1.582185	2.865553	1.515173
O	0.703777	4.498497	0.284057
C	1.983226	4.698465	-0.272371
H	2.697820	4.980259	0.492764
H	2.335796	3.800341	-0.766221
H	1.878440	5.499133	-0.991554

$Me_2\underline{C}CO_2Me^{\bullet}\_C_2H_4\_TS$

22

E: -424.876023599 ; H: -424.673822 ; G: -424.724701

C	-1.520053	0.158987	0.236216
H	-0.924001	-0.728729	0.106827
H	-2.586985	0.051471	0.135165
C	-0.959585	1.339234	0.589967
H	-1.549988	2.240796	0.544966
H	0.108259	1.468376	0.516902
C	-0.871927	1.408751	2.739895
C	-2.294290	1.249807	3.133130
H	-2.646692	0.259202	2.865628
H	-2.405154	1.357986	4.212002
H	-2.934247	1.986661	2.664459
C	0.048499	0.291980	3.062126
H	1.028295	0.444756	2.627124
H	0.179152	0.210164	4.141336
H	-0.358980	-0.649854	2.710032
C	-0.276953	2.738085	2.763425
O	0.905776	2.960063	2.731002
O	-1.182238	3.715615	2.780227
C	-0.660179	5.025857	2.741267
H	-1.510394	5.691686	2.793290
H	0.002060	5.202481	3.580603
H	-0.112698	5.196049	1.821197

Me$_2$C(CO$_2$Me)CH$_2\underline{C}$H$_2$

22

E: -424.909828621 ; H: -424.705162 ; G: -424.754975

C	-1.552609	0.078292	0.373852
H	-0.894702	-0.728105	0.101646
H	-2.612115	-0.104155	0.329264
C	-1.023663	1.354772	0.880640
H	-1.677470	2.172391	0.589880
H	-0.043471	1.547308	0.449244
C	-0.877547	1.403694	2.422621
C	-2.237413	1.261336	3.082222
H	-2.640439	0.275370	2.875678
H	-2.160502	1.371472	4.159792
H	-2.936427	2.002985	2.713165
C	0.059886	0.320074	2.901560
H	1.032687	0.402243	2.429703
H	0.206653	0.379196	3.974352
H	-0.356902	-0.653705	2.666654
C	-0.289937	2.749474	2.765569
O	0.790065	2.929584	3.245546
O	-1.108198	3.745512	2.457642
C	-0.624398	5.045989	2.726464
H	-1.409664	5.726908	2.429887
H	-0.409265	5.165094	3.781712
H	0.275610	5.246196	2.157193

nBu•

13

E: -157.735052073 ; H: -157.608699 ; G: -157.645412

C	-1.600314	-0.382431	-0.023699
H	-1.260990	-1.414643	-0.024378
H	-1.273684	0.073105	-0.953290
H	-2.686805	-0.394621	-0.025888
C	-1.066988	0.364755	1.174892
H	-1.398395	1.399945	1.146480
H	0.019649	0.389440	1.145447
C	-1.513806	-0.263748	2.493342
H	-1.173455	-1.297629	2.508257
H	-2.602102	-0.290016	2.504608
C	-1.011125	0.454210	3.676985
H	-1.535681	1.310521	4.065988
H	-0.029766	0.250377	4.070881

nBu•_C_2H_4_TS

19

E: -236.288970425 ; H: -236.105148 ; G: -236.149242

C	-1.476609	-0.115396	0.219147
H	-0.879818	-1.012480	0.213306
H	-2.540868	-0.236746	0.102917
C	-0.929639	1.089358	0.443614
H	-1.513511	1.986836	0.324827
H	0.140085	1.216683	0.434808
C	-0.860351	1.570311	2.662065
H	-0.229515	0.756114	2.982422
H	-1.907489	1.441778	2.886302
C	-0.301371	2.941112	2.701771
H	0.674292	2.960058	2.218385
H	-0.939393	3.620676	2.138651
C	-0.147722	3.485405	4.116006
H	-1.117726	3.478136	4.607672
H	0.490637	2.815589	4.687534
C	0.426804	4.882191	4.133836
H	-0.209505	5.574268	3.589097
H	0.530135	5.257487	5.147212
H	1.409405	4.907051	3.670847

nHex•

19

E: -236.342123534 ; H: -236.155230 ; G: -236.198855

C	-1.466558	-0.085932	0.559712
H	-0.841562	-0.962597	0.584489
H	-2.532731	-0.236083	0.532119
C	-0.900761	1.253080	0.796525
H	-1.499580	2.007376	0.288874
H	0.103215	1.314772	0.380723
C	-0.829505	1.620814	2.276981
H	-0.228899	0.879357	2.800493
H	-1.828099	1.564874	2.706030
C	-0.254002	2.998714	2.507720
H	0.745094	3.049447	2.075783
H	-0.853188	3.733611	1.970934

C	-0.187860	3.380221	3.968820
H	-1.186438	3.322788	4.398552
H	0.414084	2.647493	4.503137
C	0.379804	4.761569	4.191059
H	-0.219219	5.516581	3.689069
H	0.411961	5.015344	5.246107
H	1.392082	4.837003	3.803296

$MeOCO_2CH_2$•

11

E: -342.925032712 ; H: -342.834098 ; G: -342.872680

C	-1.110474	-0.236526	-0.084034
O	-0.079412	-0.291632	-0.675301
O	-2.307148	-0.051886	-0.635297
O	-1.274473	-0.344888	1.209414
C	-0.081254	-0.554082	1.945446
H	-0.380151	-0.611959	2.981731
H	0.394532	-1.477918	1.640460
H	0.604028	0.271622	1.799383
C	-2.363829	0.093654	-1.978129
H	-1.482167	-0.103916	-2.554558
H	-3.363474	0.136955	-2.359488

$MeOCO_2CH_2$•_C_2H_4_TS

17

E: -421.481613040 ; H: -421.332848 ; G: -421.378256

C	-1.258657	1.529425	0.109775
H	-2.030582	2.016219	-0.462026
H	-0.266326	1.945105	0.048797
C	-1.532886	0.496985	0.921725
H	-0.734536	-0.051368	1.393949
H	-2.492349	0.007345	0.888570
C	-2.070460	1.337404	2.949324
H	-3.079451	1.672112	2.789092
H	-1.882744	0.478831	3.567957
O	-1.148128	2.314700	3.171565
O	-0.380204	4.288453	2.909396
C	-1.432721	3.529044	2.722300
O	-2.474306	3.872901	2.257510
C	-0.544674	5.641382	2.525407
H	0.390960	6.129857	2.755597
H	-0.752538	5.714109	1.464853
H	-1.351833	6.099986	3.083518

$MeOCO_2CH_2CH_2CH_2$•

17

E: -421.535277167 ; H: -421.384014 ; G: -421.431439

C	-0.343616	-0.050539	1.366143
H	0.309433	-0.112585	0.514219
H	-0.130880	-0.683147	2.211585
C	-1.605833	0.703061	1.298072
H	-2.413938	0.057493	0.941228

H	-1.528821	1.522492	0.588527
C	-2.006788	1.237150	2.649309
H	-2.074632	0.435514	3.375838
H	-1.296836	1.973717	3.002570
O	-3.249751	1.925460	2.591905
O	-5.383007	1.955514	2.534178
C	-4.328730	1.176186	2.626459
O	-4.356159	-0.013554	2.732767
C	-6.628064	1.284574	2.571368
H	-7.382021	2.055471	2.501364
H	-6.717844	0.598440	1.737843
H	-6.740765	0.738459	3.499985

MMA_2_Bu•_TS

43

E: -849.258642573 ; H: -848.856370 ; G: -848.935462

C	-2.953907	1.810920	0.899304
C	-3.086859	0.801624	1.955126
O	-2.169402	0.379493	2.604672
O	-4.332260	0.371473	2.127796
C	-4.498501	-0.620415	3.117734
H	-5.553372	-0.856757	3.132352
H	-3.922457	-1.506341	2.876645
H	-4.186748	-0.252187	4.088151
C	-1.600867	2.403431	0.700884
H	-0.852360	1.687601	1.026856
H	-1.444883	2.591239	-0.356023
C	-1.370668	3.726716	1.457610
C	-2.276685	4.820576	0.918234
H	-2.052850	5.772319	1.391112
H	-2.164428	4.938565	-0.153956
H	-3.311842	4.579471	1.135154
C	-1.586065	3.561976	2.944122
H	-1.364061	4.485462	3.467101
H	-2.622297	3.305040	3.140038
H	-0.956963	2.780722	3.351861
C	0.063274	4.132272	1.214141
O	0.891660	4.289049	2.060614
O	0.320863	4.304670	-0.075116
C	1.644103	4.686726	-0.392301
H	1.890728	5.634533	0.071504
H	2.351196	3.936380	-0.059202
H	1.680603	4.779511	-1.468595
C	-3.998356	2.106457	0.108350
H	-4.983540	1.741673	0.332392
H	-3.917654	2.886569	-0.628875
C	-4.090154	0.624190	-1.760182
C	-5.361815	1.015065	-2.402215
H	-4.051788	-0.293593	-1.196667
H	-3.161678	0.931179	-2.213980
H	-6.190039	0.870080	-1.710853
H	-5.345631	2.073863	-2.656020

C	-5.649980	0.221481	-3.671835
C	-6.956777	0.624656	-4.312157
H	-5.665920	-0.839011	-3.432488
H	-4.833091	0.365837	-4.374725
H	-7.146943	0.056383	-5.217194
H	-7.791720	0.458532	-3.637138
H	-6.954215	1.678115	-4.578115

MMA$_2$_Bu•

43

E: -849.319647023 ; H: -848.912914 ; G: -848.988396

C	-2.698725	1.294656	0.652512
C	-2.858399	0.244056	1.627091
O	-1.955414	-0.255788	2.252920
O	-4.126571	-0.144169	1.779145
C	-4.333598	-1.170476	2.723808
H	-5.396875	-1.366628	2.728285
H	-3.791973	-2.067158	2.444713
H	-4.009513	-0.858489	3.710081
C	-1.341908	1.836286	0.434287
H	-0.610562	1.075675	0.692142
H	-1.226876	2.086131	-0.616660
C	-1.015477	3.101680	1.261362
C	-1.934728	4.256866	0.898431
H	-1.620349	5.164405	1.405639
H	-1.938233	4.449203	-0.168533
H	-2.948751	4.038446	1.216498
C	-1.100812	2.821653	2.743546
H	-0.788909	3.690269	3.312298
H	-2.124192	2.585495	3.015315
H	-0.474539	1.984956	3.026707
C	0.400628	3.504089	0.921158
O	1.293542	3.613842	1.706925
O	0.555385	3.744613	-0.372852
C	1.853911	4.137980	-0.771210
H	2.145979	5.053911	-0.271372
H	2.575205	3.363582	-0.538994
H	1.807606	4.295922	-1.839449
C	-3.834041	1.722776	-0.193006
H	-4.738428	1.811177	0.403236
H	-3.619187	2.699396	-0.615574
C	-4.102218	0.746026	-1.335721
C	-5.258393	1.184989	-2.202796
H	-4.306635	-0.237554	-0.920089
H	-3.204220	0.649722	-1.943002
H	-6.154915	1.269818	-1.589854
H	-5.061026	2.183204	-2.592352
C	-5.525824	0.243071	-3.354857
C	-6.693858	0.675883	-4.207916
H	-5.709214	-0.756089	-2.964082
H	-4.631153	0.168177	-3.970403
H	-6.863768	-0.012191	-5.030144

H	-7.609113	0.723925	-3.624251
H	-6.525296	1.661543	-4.633143

MMA$_2$_Bu•_C$_2$H$_4$_TS
49
E: -927.866241571 ; H: -927.402152 ; G: -927.481257

C	-3.342389	0.681104	2.142063
O	-2.795420	0.415421	3.179344
O	-4.652624	0.497079	1.967886
C	-5.343920	-0.035616	3.075978
H	-6.372621	-0.149508	2.763080
H	-4.933707	-0.997290	3.361737
H	-5.288100	0.635102	3.925612
C	-1.236216	1.525258	1.082464
H	-0.800807	0.853975	1.815123
H	-0.733691	1.349049	0.135748
C	-0.890791	2.950893	1.522175
C	-1.662942	3.377990	2.752447
H	-1.349071	4.358169	3.089977
H	-2.732201	3.409350	2.571082
H	-1.486459	2.662422	3.547065
C	0.603501	2.983805	1.836381
H	0.927388	3.988773	2.087773
H	0.806620	2.340102	2.686127
H	1.189028	2.632673	0.992783
C	-1.056427	3.907042	0.364250
O	-0.934120	3.609806	-0.788492
O	-1.279384	5.151967	0.754421
C	-1.373284	6.110630	-0.280392
H	-0.453832	6.149384	-0.852387
H	-2.194306	5.875040	-0.947751
H	-1.549654	7.060376	0.204482
C	-3.511825	1.749444	-0.155935
H	-2.872937	1.937225	-1.014473
H	-4.258697	1.020735	-0.462205
C	-4.256057	3.027758	0.196669
C	-5.120163	3.496475	-0.952132
H	-3.564412	3.821129	0.462050
H	-4.875642	2.853592	1.072462
H	-4.494210	3.655419	-1.829983
H	-5.820530	2.705599	-1.218973
C	-5.889653	4.761730	-0.650693
C	-6.757049	5.203832	-1.805081
H	-5.190989	5.555796	-0.393153
H	-6.507069	4.604168	0.231734
H	-7.303366	6.112623	-1.572326
H	-6.159264	5.396097	-2.691952
H	-7.484593	4.438648	-2.061589
C	-2.675209	1.151810	0.930623
C	-2.463980	-0.781061	0.094667
H	-2.124363	-0.325476	-0.821935
H	-3.523663	-0.972422	0.150951

C	-1.605827	-1.551962	0.813027
H	-1.949297	-2.160401	1.632099
H	-0.543159	-1.516546	0.642195

MMA_2_Bu_C_2H_4•
49
E: -927.893086519 ; H: -927.426891 ; G: -927.505462

C	-3.365064	0.629233	2.072420
O	-2.869710	0.531843	3.156236
O	-4.649595	0.376252	1.857001
C	-5.397993	-0.011841	2.991178
H	-6.408902	-0.172080	2.643811
H	-5.001831	-0.925363	3.418955
H	-5.382506	0.765782	3.745593
C	-1.226364	1.473904	1.068272
H	-0.794855	0.848567	1.844195
H	-0.642420	1.302219	0.168297
C	-0.974809	2.919637	1.487066
C	-1.786684	3.362406	2.686352
H	-1.539426	4.380088	2.962904
H	-2.854818	3.308851	2.512328
H	-1.561326	2.711443	3.522149
C	0.507612	3.017473	1.858191
H	0.780003	4.038973	2.104419
H	0.702292	2.396363	2.726348
H	1.138463	2.677913	1.043290
C	-1.098204	3.847889	0.300190
O	-0.877595	3.536344	-0.833838
O	-1.387462	5.093269	0.646944
C	-1.423811	6.030567	-0.410243
H	-0.445618	6.131180	-0.866074
H	-2.134910	5.727331	-1.169242
H	-1.725810	6.969826	0.030990
C	-3.467233	1.688083	-0.223847
H	-2.817295	1.911406	-1.065753
H	-4.225875	1.002143	-0.593441
C	-4.178134	2.952803	0.206346
C	-5.074773	3.482384	-0.891636
H	-3.477974	3.733082	0.481382
H	-4.780534	2.760713	1.091252
H	-4.482204	3.653091	-1.790348
H	-5.808505	2.721199	-1.155002
C	-5.791150	4.757902	-0.512284
C	-6.693934	5.273835	-1.607277
H	-5.057024	5.519483	-0.254653
H	-6.374603	4.584844	0.390328
H	-7.198210	6.188730	-1.312033
H	-6.131084	5.485192	-2.512450
H	-7.457820	4.543752	-1.860295
C	-2.623474	0.921905	0.792722
C	-2.405069	-0.492218	0.157100
H	-1.969460	-0.310467	-0.821190

H	-3.395246	-0.915773	-0.001076
C	-1.571212	-1.424470	0.929162
H	-1.950257	-1.891024	1.822345
H	-0.539168	-1.593750	0.678640

MMA$_2$_Bu•_frag_TS

43

E: -849.278830532 ; H: -848.875043 ; G: -848.950856

C	-2.102196	1.013429	0.092375
C	-2.260729	-0.067243	1.071443
O	-1.403800	-0.454220	1.818402
O	-3.481354	-0.594357	1.056733
C	-3.702276	-1.641647	1.977410
H	-4.722943	-1.964621	1.827377
H	-3.021252	-2.464546	1.795037
H	-3.568615	-1.292744	2.994744
C	-0.932962	1.697948	0.120297
H	-0.115635	1.317300	0.708663
H	-0.701173	2.384012	-0.677787
C	-1.184627	3.401944	1.527933
C	-2.274732	4.180201	0.894803
H	-2.442608	5.105617	1.447496
H	-2.048529	4.452709	-0.128168
H	-3.202046	3.618581	0.918176
C	-1.480315	2.709279	2.801101
H	-1.568015	3.436987	3.608949
H	-2.424449	2.178891	2.738614
H	-0.696537	2.013318	3.068982
C	0.195583	3.832934	1.348609
O	1.118277	3.467108	2.028071
O	0.353246	4.669067	0.323552
C	1.678115	5.099518	0.094224
H	2.065818	5.625047	0.959075
H	2.325823	4.258625	-0.124883
H	1.635748	5.766799	-0.755304
C	-3.198247	1.323584	-0.865849
H	-4.150344	1.368484	-0.342282
H	-3.019149	2.307015	-1.291448
C	-3.309969	0.308439	-1.992315
C	-4.455306	0.610437	-2.931022
H	-3.437939	-0.684527	-1.568917
H	-2.374397	0.290854	-2.548170
H	-5.386740	0.631089	-2.365971
H	-4.330642	1.610695	-3.345239
C	-4.581265	-0.384854	-4.061259
C	-5.736143	-0.077552	-4.984668
H	-4.701598	-1.383770	-3.646174
H	-3.653237	-0.402555	-4.629829
H	-5.812214	-0.804713	-5.786999
H	-6.679789	-0.082256	-4.445792
H	-5.624512	0.903044	-5.439540

MMA_2_Bu
27
E: -502.961462923 ; H: -502.704901 ; G: -502.759680

C	-2.609460	1.518181	0.172494
C	-2.774321	0.649520	1.365196
O	-1.883722	0.269205	2.068186
O	-4.043235	0.341734	1.587327
C	-4.287239	-0.474701	2.715180
H	-5.354756	-0.641306	2.740439
H	-3.763979	-1.419028	2.623490
H	-3.965618	0.021241	3.623188
C	-1.397577	1.992108	-0.053401
H	-0.579885	1.741098	0.600210
H	-1.198669	2.640500	-0.890458
C	-3.786434	1.799582	-0.698161
H	-4.636285	2.076813	-0.080447
H	-3.553920	2.653877	-1.327577
C	-4.176030	0.622660	-1.574401
C	-5.405438	0.904043	-2.406219
H	-4.352284	-0.250745	-0.951420
H	-3.340805	0.374603	-2.226675
H	-6.236199	1.152562	-1.746469
H	-5.232252	1.787355	-3.020454
C	-5.802602	-0.251408	-3.295956
C	-7.027359	0.044813	-4.128121
H	-5.982290	-1.131614	-2.681300
H	-4.968830	-0.503071	-3.948929
H	-7.294462	-0.795651	-4.761168
H	-7.884004	0.267792	-3.497815
H	-6.863485	0.903761	-4.773037

MMA_2_Me_2(CN)C•_TS
41
E: -902.210316941 ; H: -901.836228 ; G: -901.911925

C	-2.934052	1.803534	0.809940
C	-3.036692	0.783944	1.860408
O	-2.090254	0.296626	2.415699
O	-4.285931	0.434441	2.139011
C	-4.433455	-0.571569	3.120029
H	-5.496197	-0.742125	3.218085
H	-3.936582	-1.484524	2.812915
H	-4.019750	-0.246873	4.067432
C	-1.602088	2.434467	0.614215
H	-0.829212	1.720088	0.882493
H	-1.475751	2.696324	-0.429951
C	-1.392485	3.718152	1.447202
C	-2.326425	4.821349	0.978025
H	-2.118431	5.747731	1.505118
H	-2.223751	5.005686	-0.085689
H	-3.355714	4.549559	1.186767
C	-1.588529	3.466252	2.923933
H	-1.382465	4.365329	3.493429

H	-2.617077	3.176827	3.116638
H	-0.937446	2.679982	3.284939
C	0.030661	4.166690	1.212017
O	0.857937	4.314293	2.060741
O	0.276517	4.388205	-0.071286
C	1.592485	4.801350	-0.382809
H	1.826493	5.738220	0.108634
H	2.311554	4.051825	-0.074590
H	1.622002	4.927768	-1.455774
C	-3.999997	2.084729	0.016879
H	-4.986029	1.761877	0.298931
H	-3.939052	2.910738	-0.671694
C	-4.010096	0.638276	-1.655929
C	-2.683547	0.835589	-2.300047
H	-2.533749	1.858378	-2.625114
H	-1.893828	0.559962	-1.610421
H	-2.595217	0.193241	-3.175660
C	-4.254109	-0.660795	-0.973271
H	-4.316692	-1.460792	-1.710158
H	-3.426404	-0.893907	-0.312176
H	-5.174912	-0.657044	-0.403557
C	-5.115672	1.220897	-2.307173
N	-6.016731	1.729936	-2.802239

MMA_2_Me_2(CN)C•_product

41

E: -902.250793969 ; H: -901.873651 ; G: -901.946999

C	-2.845931	1.645327	0.784480
C	-3.019970	0.720610	1.884804
O	-2.116631	0.301939	2.563789
O	-4.287100	0.366434	2.086075
C	-4.499293	-0.564650	3.126067
H	-5.562412	-0.759013	3.143448
H	-3.955283	-1.482956	2.937489
H	-4.180021	-0.157090	4.078235
C	-1.531483	2.307179	0.650168
H	-0.760618	1.633320	1.014181
H	-1.341527	2.522110	-0.395294
C	-1.395953	3.641583	1.427196
C	-2.382040	4.678644	0.916079
H	-2.196577	5.640847	1.384194
H	-2.309026	4.806651	-0.158018
H	-3.396235	4.385509	1.166176
C	-1.575635	3.441170	2.913149
H	-1.411363	4.372985	3.442521
H	-2.586882	3.108678	3.123348
H	-0.886532	2.702854	3.303023
C	0.006639	4.142219	1.165568
O	0.844593	4.312596	1.998901
O	0.217279	4.379430	-0.120592
C	1.511976	4.838220	-0.458739
H	1.722865	5.782083	0.029633

H	2.262428	4.113579	-0.166371
H	1.514718	4.966789	-1.531812
C	-3.932760	1.900114	-0.188812
H	-4.897721	1.851295	0.304117
H	-3.813039	2.897324	-0.600143
C	-3.963310	0.914314	-1.385363
C	-2.675864	0.966095	-2.192343
H	-2.434519	1.980090	-2.491557
H	-1.859882	0.572408	-1.596539
H	-2.764791	0.358019	-3.085260
C	-4.242826	-0.513490	-0.947224
H	-4.302387	-1.165790	-1.811476
H	-3.431166	-0.864252	-0.318895
H	-5.170558	-0.586159	-0.393832
C	-5.059248	1.353702	-2.244581
N	-5.914589	1.701919	-2.913850

Copolymerisation of different methacrylic functional oligomers with ethylene

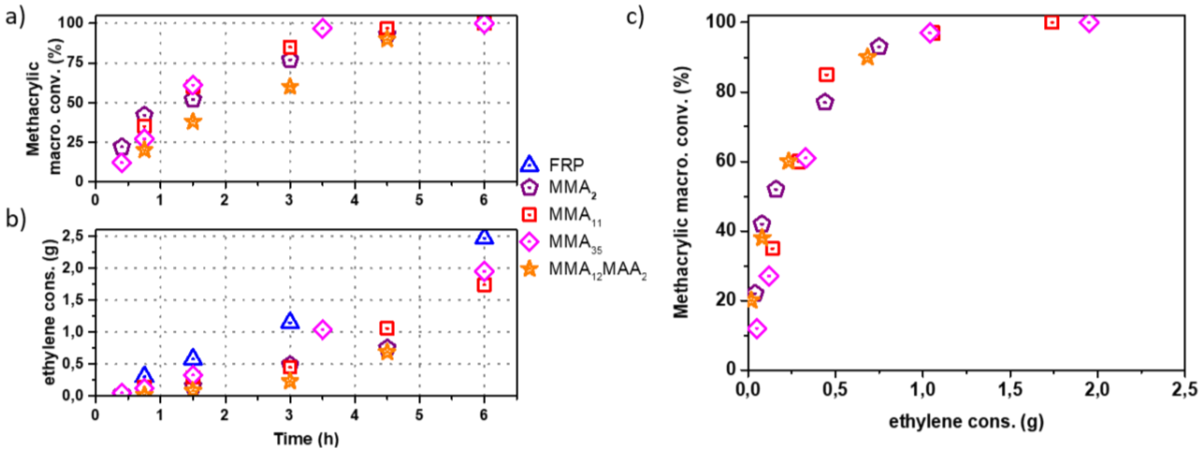

Figure S8. a) Methacrylic oligomer conversion and b) ethylene consumption versus polymerisation time. c) Methacrylic oligomer conversion versus ethylene consumption in the presence of different oligomers (Table 3).

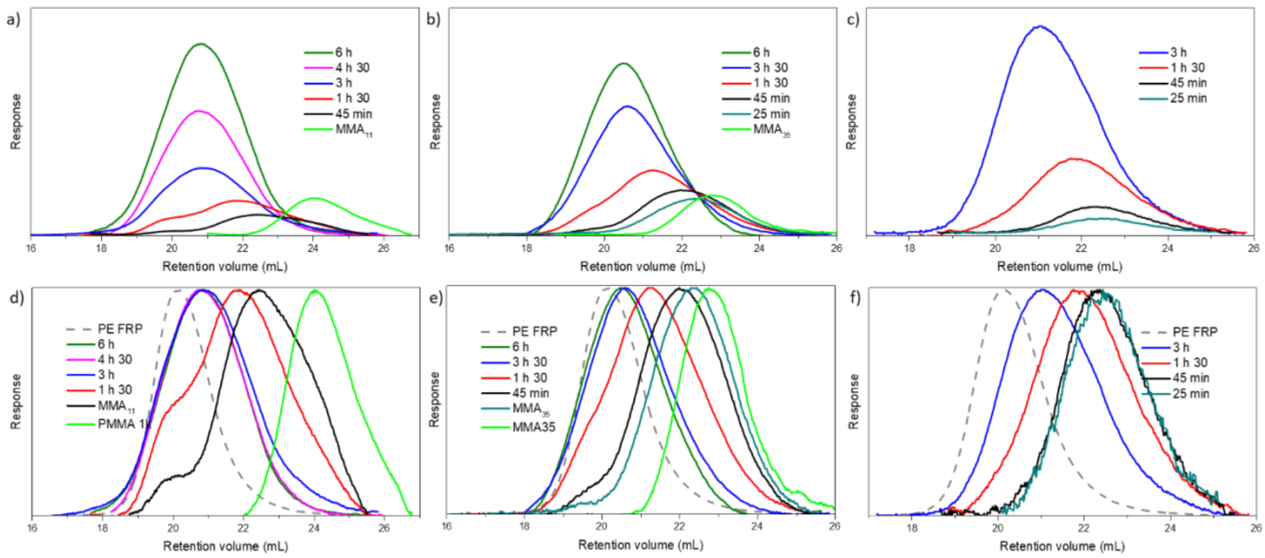

Figure S9. Molar mass distributions of PE obtained during copolymerisation of ethylene and a) MMA_{11} b) MMA_{35} and c) $MMA_{12}MAA_{2}$ at 70°C and 80 bar of ethylene pressure after the indicated polymerisation time (Table 3 and run 3 Table 2). a-c) are normalised by yields whereas d-f) are normalised by areas.

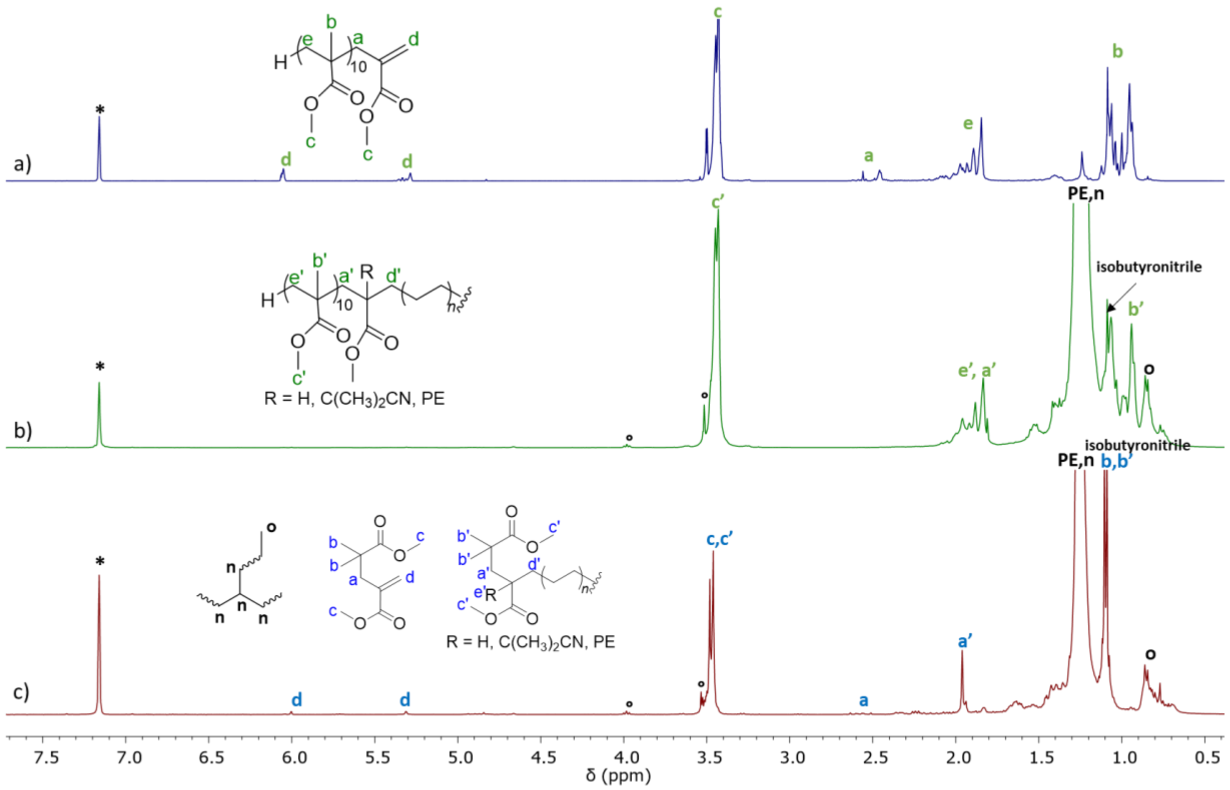

Figure S10. ^{1}H NMR spectra (in TCE/C_6D_6 at 90 °C) of a) MMA_{11}, PE synthesised in the presence of b) MMA_{11} and c) MMA_{2} (cf. Table 3 run 4 and Table 2 run 8, respectively). *NMR solvent benzene, ° transfer to polymerisation solvent (DMC).

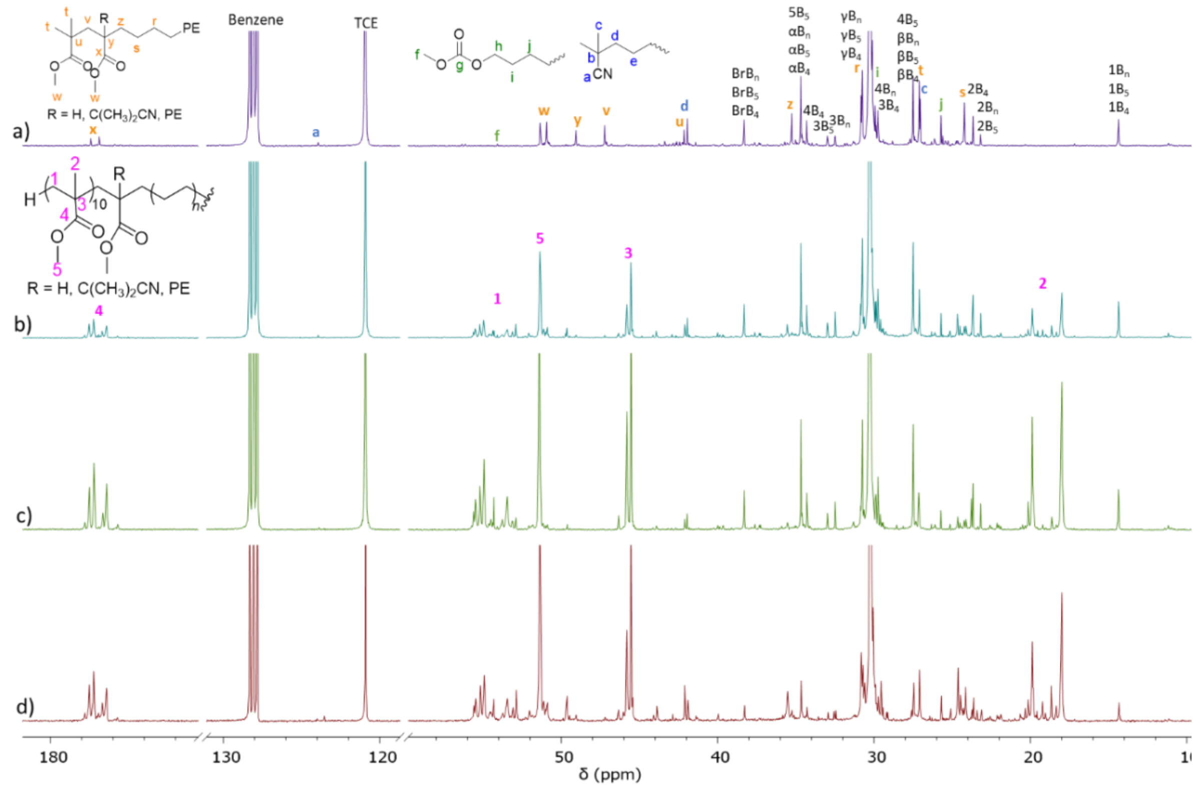

Figure S11. ^{13}C NMR spectra (in TCE/C_6D_6 at 90 °C) of PE synthesised in the presence of a) MMA_2 (run 4 in Table 2), b) MMA_{11} (run 4 in Table 3), c) MMA_{35} (run 9 in Table 3) and c) MMA_{11} with a molar ratio $[MMA_{11}]/[AIBN]$ of 3 (run 5 in Table 4).

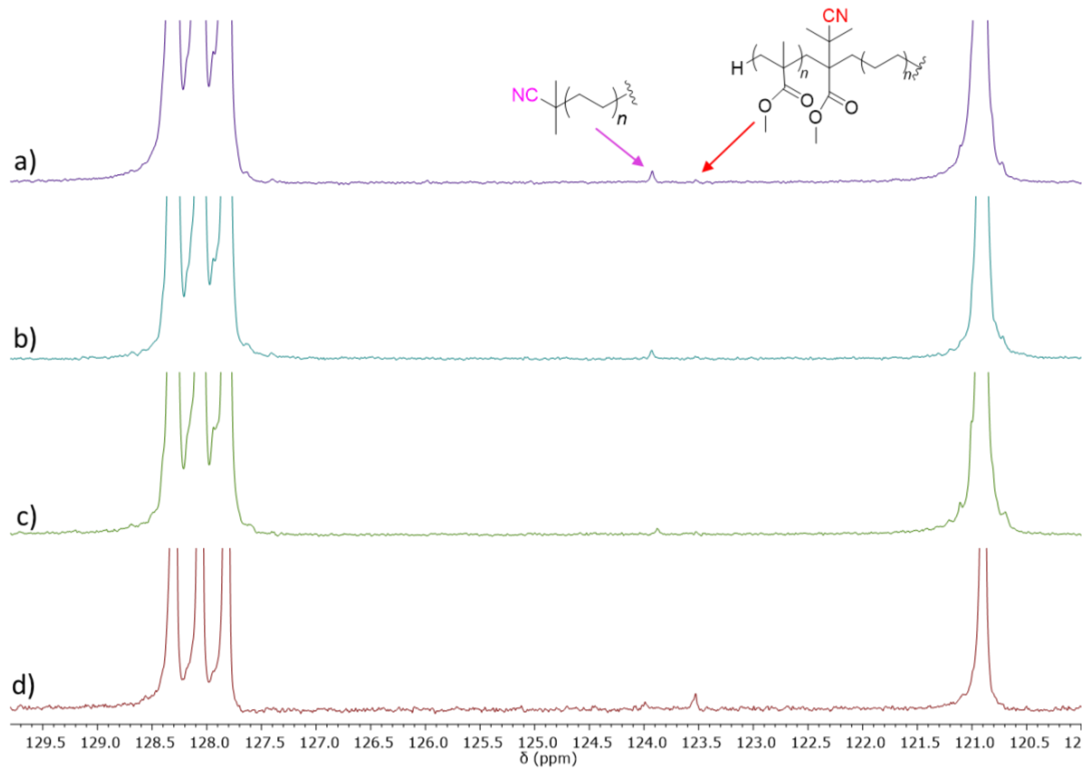

Figure S12: Expansion between 120 and 130 ppm of ^{13}C NMR spectrum (in TCE/C_6D_6 at 90 °C) of PE synthesised in the presence of a) MMA_2 (run 4 in Table 2), b) MMA_{11} (run 4 in Table 3), c) MMA_{35} (run 9 in Table 3) and d) MMA_{11} with a molar ratio $[MMA_{11}]/[AIBN]$ of 3 (run 5 in Table 4).

Calculation of the chain transfer constant C_s' (= k_{tr}/k_p) for methacrylic oligomers [14–16]

For clarity all methacrylic oligomers (MMA_2, MMA_{11}, MMA_{35} and $MMA_{12}MAA_2$) will be abbreviated as MMA_n.

Transfer with vinyl terminated PMMA chain transfer:

1. $PE^{\bullet} + MMA_n \overset{k_{tr}}{\rightarrow} PE\text{-}MMA_n$,

The consumption of MMA_n over time t based on reaction 1 is

$$-\frac{d[MMA_n]}{dt} = k_{tr} \cdot [PE^{\bullet}] \cdot [MMA_n], \qquad (1)$$

With k_{tr}, $[MMA_n]$ and $[PE°]$ are transfer rate, concentration in MMA_n and radicals, respectively. An integration from the polymerization time t = 0 to t = t and the respective concentrations of $[MMA_n]$, $[MMA_n]_0$ to $[MMA_n]_t$, gives:

$$-\int_{[MMA_n]_0}^{[MMA_n]_t} \frac{d[MMA_n]}{dt} = \int_{t=0}^{t} k_{tr} \cdot [PE^{\bullet}] \cdot dt \longrightarrow \ln\left(\frac{[MMA_n]_0}{[MMA_n]_t}\right) = k_{tr} \cdot [PE^{\bullet}] \cdot t \qquad (2)$$

Propagation:

2. $PE^{\bullet} + C_2H_4 \overset{k_p}{\rightarrow} (C_2H_4)PE$

Based on Reaction 2, the generation of ethylene units in PE is given by

$$\frac{d[(C_2H_4)PE]}{dt} = k_p \cdot [PE^{\bullet}] \cdot [C_2H_4], \qquad (3)$$

With k_p, $[C_2H_4]$, $[PE°]$ and $[(C_2H_4)PE]$ are propagation rate, concentration in monomer, radicals, ethylene conversion respectively. The ethylene pressure is kept constant during the polymerization:

$$[(C_2H_4)PE]_t = k_p \cdot [PE^{\bullet}] \cdot [C_2H_4] \cdot t\,. \qquad (4)$$

Equation 2 divided by Equation 4 gives

$$\ln\left(\frac{[MMA_n]_0}{[MMA_n]_t}\right) = \frac{k_{tr}}{k_p} \cdot \frac{[(C_2H_4)PE]_t}{[C_2H_4]} = C'_s \cdot \frac{[(C_2H_4)PE]_t}{[C_2H_4]}\,. \qquad (5)$$

$(C_2H_4)PE$ and C_2H_4 are both referring to ethylene species with identical molar masses in the same system, hence volume of solvent. The fraction in Equation 5 can thus be also given in terms of mass in grams.

(6)

$$C'_s = \frac{k_{\mathrm{tr}}}{k_{\mathrm{p}}} = \ln\left(\frac{100}{\mathrm{X}}\right) \cdot \frac{m(\mathrm{C_2H_4})}{m\big((\mathrm{C_2H_4})\mathrm{PE}\big)}$$

$m(C_2H_4)$ is constant during the polymerization and is the quantity of ethylene soluble in the reaction medium. $m((C_2H_4)PE)$ is the gravimetrically determined ethylene conversion. X is the conversion of MMA_n. The solubility of ethylene in DMC was determined experimentally and a value of 327 g L^{-1} at 70 °C and 80 bar.[16,17] The chain transfer constant is determined by linear regression from Equation 7.

$$\ln\left(\frac{100}{\mathrm{X}}\right) = \frac{m\big((\mathrm{C_2H_4})\mathrm{PE}\big)}{m(\mathrm{C_2H_4})} C'_s \qquad (7)$$

Table S1. Transfer constant to MMA_n C_s' values at 80 bar and 70 °C for different MMA_n.

MMA_n	C_s'	R^2	error
MMA_2	55.6	0.993	2.0
MMA_{11} ($[MMA_{11}]/[AIBN]=1$)	56.4	0.987	3.1
MMA_{35}	52.8	0.997	1.2
$MMA_{12}MAA_2$	56.6	0.983	3.7
MMA_{11} ($[MMA_{11}]/[AIBN]=3$)	60.6	0.993	2.0

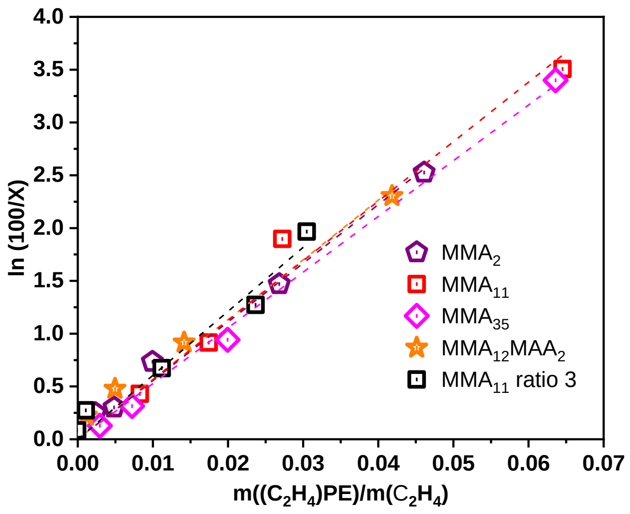

Figure S13. Linear regression of the logarithm conversion of MMA_n versus ethylene consumption to ethylene solubility ratio for the determination of the transfer constant C_s'.

Copolymerisation of ethylene with different concentrations of MMA_{11}

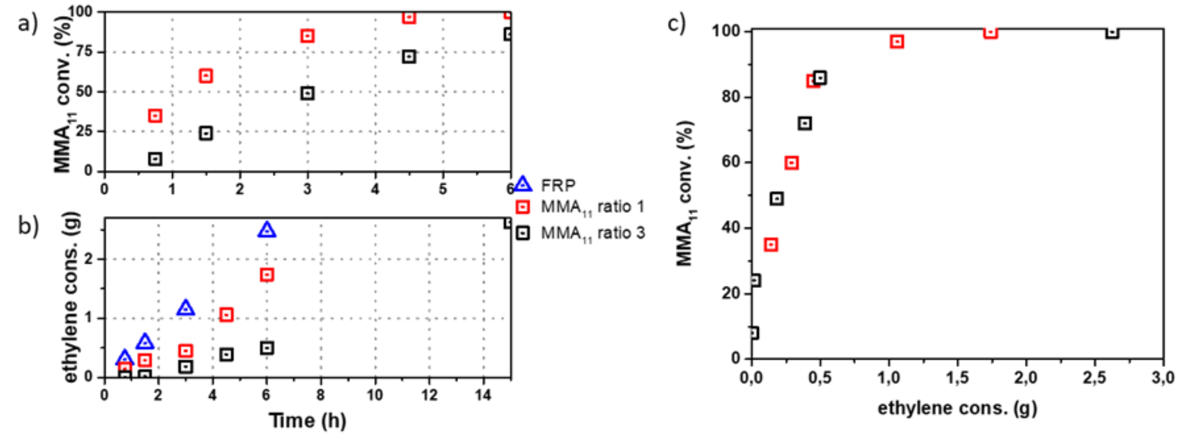

Figure S14. a) MMA_{11} conversion and b) ethylene consumption versus polymerisation time. c) MMA_{11} conversion versus ethylene consumption at different MMA_{11} concentrations (Table 4).

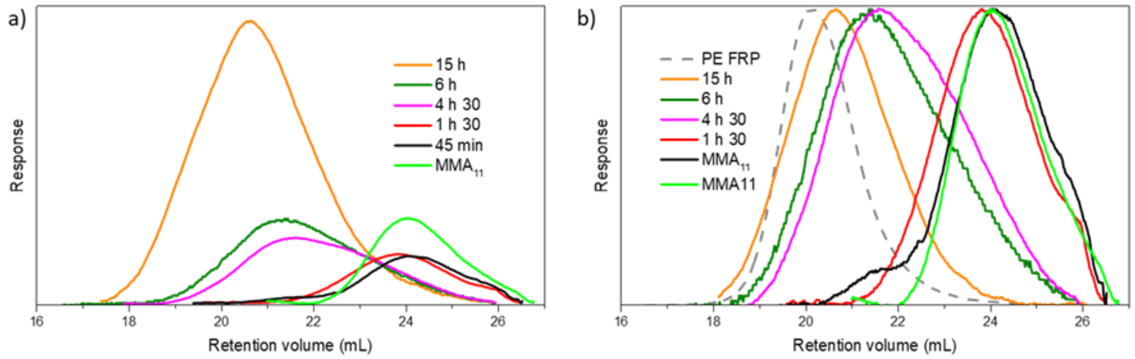

Figure S15. Molar mass distributions of PE obtained during copolymerisation of ethylene and MMA_{11} at 70 °C and 80 bar of ethylene pressure after different polymerisation times (Table 4 and run 3 Table 2), normalised by a) yields and b) areas.

DSC of the different copolymers synthesized

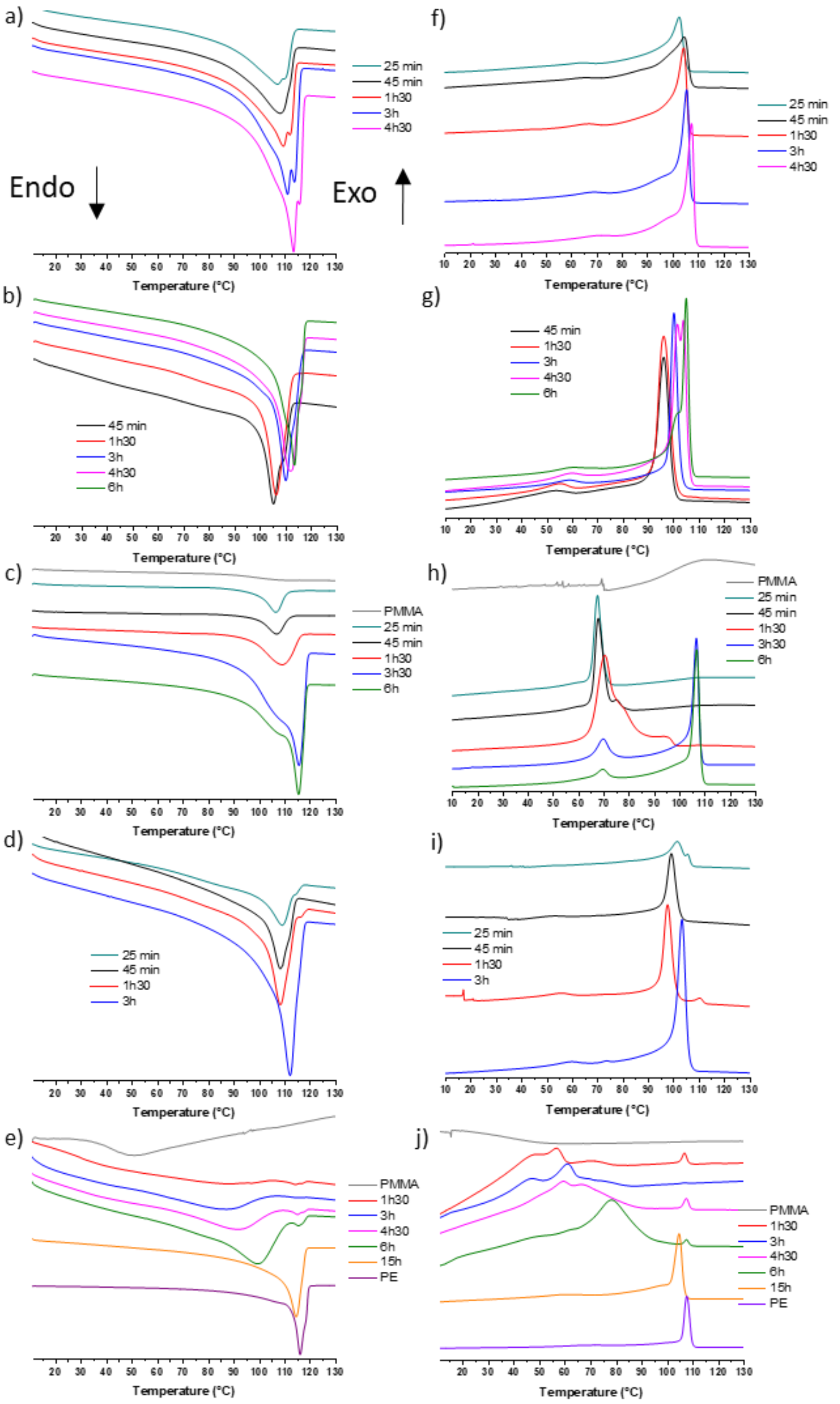

Figure S16. DSC of experiments presented in Table 1, Table 2 and Table 3. a-e) heating and f-j) cooling for the copolymerisation carried out in the presence of MMA_2, MMA_{11}, MMA_{35}, $MMA_{12}MAA_2$, $[MMA_{11}]/[AIBN] = 3$, respectively.

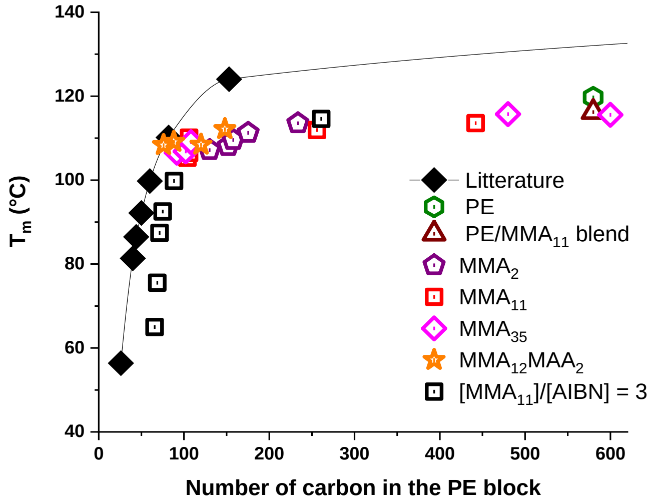

Figure S17. Comparison of melting temperatures, measured by DSC, depending on the number of carbons per PE chain, as measured by ^{1}H NMR, for the different copolymers.[18]

SAXS/WAXS analysis of the different copolymers

SAXS analysis of MMA_{11}

At high q-range ($q > 0.06$ Å^{-1}) the signal is constant around 0.002 which probably corresponds to the detector background. Otherwise, the Porod's law[19] correctly predicts the scattering intensity: $I(q) \sim Sq^{-4}$.

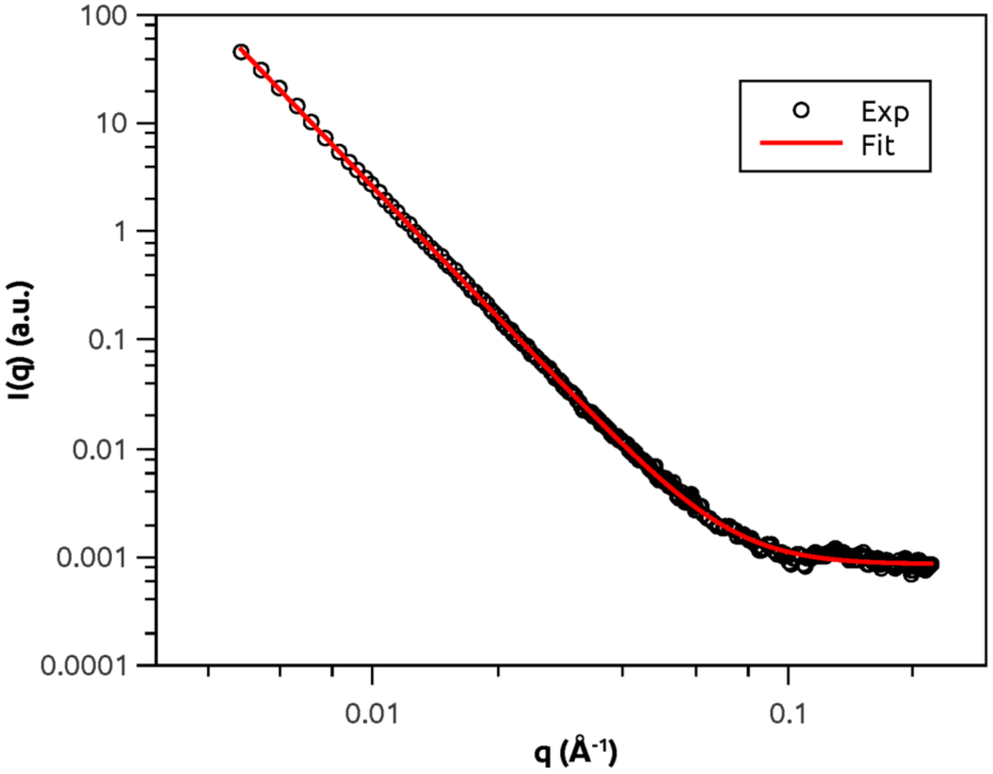

Figure S15. SAXS scattering intensities of MMA_{11}.

In the low q-range ($q < 0.04$ Å^{-1}), the scattering intensity fits the Porod law ($I(q) \propto q^{-4}$) corresponding to the amorphous PE bulk. The peak around 0.055 Å^{-1} has been fitted with a Broad Peak model[20,21] characteristic of a two-component system (with a crystalline and an amorphous phase).

$I(q) = I\,0 / (1 + (|q - q_0|\xi)^m)$.

A second broad peak of lower intensity is discernible at higher q-range (d ≈ 54 nm and ξ ≈ 6 nm).

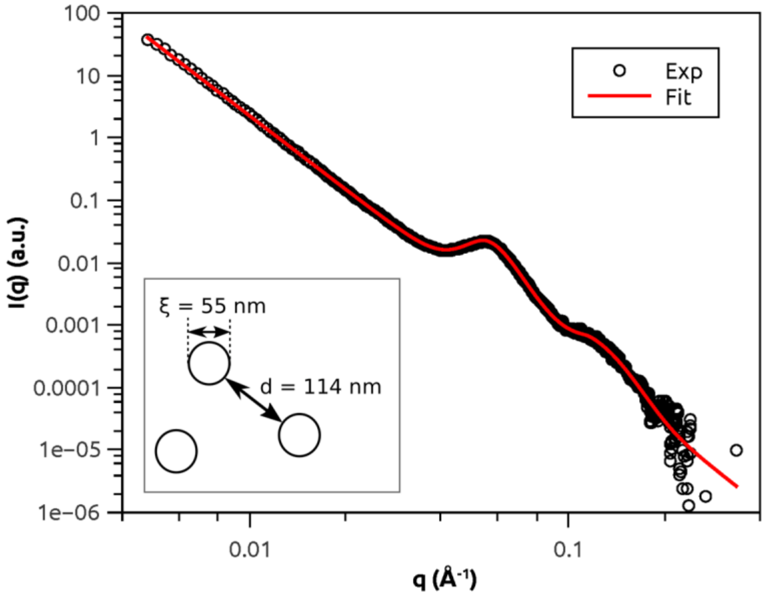

Figure S16. SAXS scattering intensities of PE bulk (run 4 in Table2).

WAXS analysis of polyethylene

The decorrelation of the diffraction peaks gives many useful information. The area of diffraction peaks and the amorphous halo (fitted using a Gaussian function) allowed estimating a crystallinity index (i_c):

i_c (%) = $A_{peak}/(A_{peak}+A_{halo})\times100$

Where A_{peak} and A_{halo} are the area of the (110) crystalline peaks (blue peak in Figure S17) and the amorphous halo (green curve in Figure S17), respectively. The characteristic domain size (τ) perpendicular to the reflecting plane of each peak can be obtained from the Scherrer equation:[22]

$\tau = K\lambda/(\beta \cos \theta)$

Where K is a dimensionless shape factor of about 0.9 under the assumption of a Gaussian line shape, λ is the X-ray wavelength, β is the line broadening at half the maximum intensity (full width at half maximum, FWHM), after subtracting the instrumental line broadening in radians and θ is the Bragg angle also in radians.

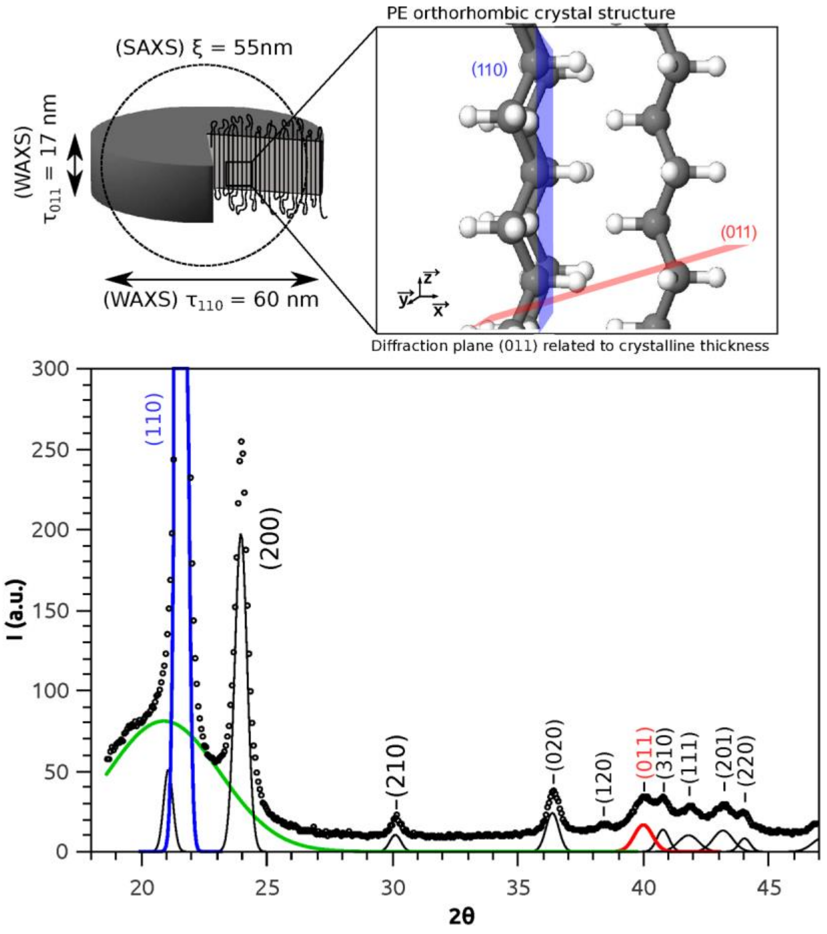

Figure S17. Diffraction pattern of PE and gaussian peak fitting for the determination of the crystallinity index and the characteristic domain size of bulk PE.[23]

Table S3: Summary of the PE crystallite dimensions for PE/MMA_{11} blend. The characteristic domain size (τ) of the (110) and (011) peaks corresponds to the diameter and the thickness of the crystallite, respectively. R = τ_{110} and L = τ_{011}

Peak position (2θ)	Area	FWHM	τ (nm)	(hkl) orthorhombic
20.89	454.66	5.30		amorphous
21.07	23.93	0.44	60.9	monoclinic
21.60	391.08	0.44	62.1	110
23.96	120.32	0.57	32.1	200
30.12	5.89	0.53	39.8	210
36.37	15.60	0.61	32.4	020
38.40	0.90	0.37	149.3	120
40.00	16.54	0.91	17.2	011
40.78	7.83	0.55	42.1	310
41.05	54.65	7.07		amorphous?
41.79	11.10	0.99	15.5	111
43.16	12.62	0.89	18.6	201
44.02	5.19	0.58	39.5	220
47.19	9.76	1.04	15.8	211

SAXS analysis of a blend of PE and MMA_{11}

Here the low q-range (q < 0.02 Å^{-1}) exhibit a q^{-4} slope, followed by a peak around 0.03 Å^{-1} (ξ ≈ 80 nm and d ≈ 200 nm), the high q-range show two wavelets characteristic of a cylindrical form factor (R = 56 nm and L = 35 nm).

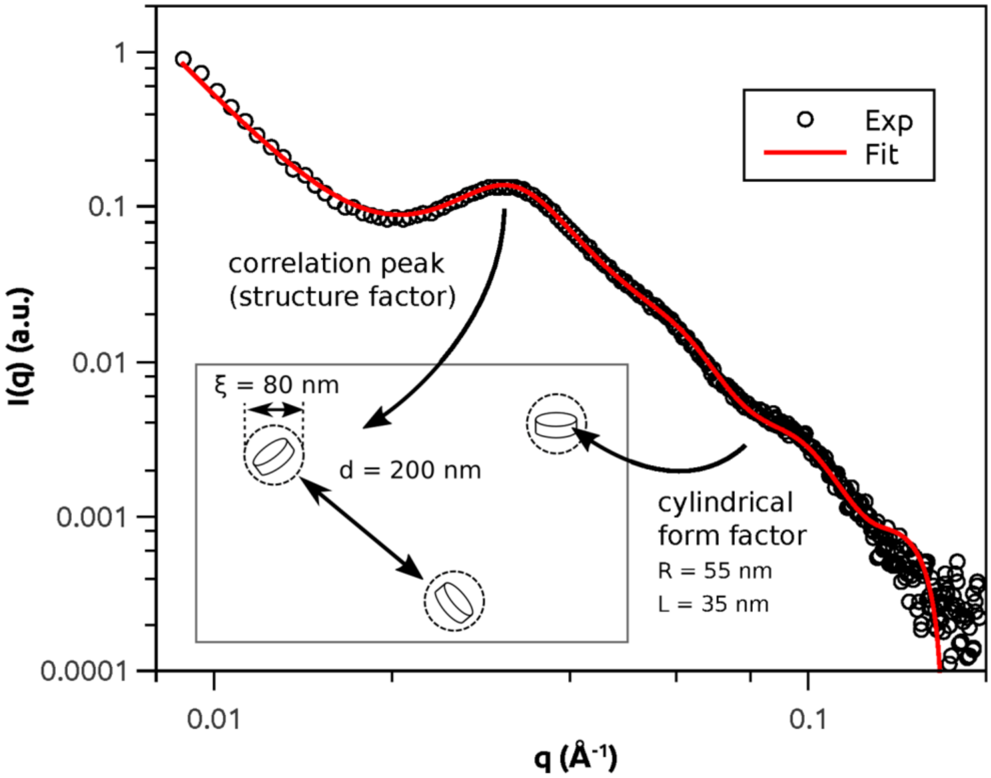

Figure S18. SAXS scattering intensities of PE/MMA_{11} blend.

WAXS analysis of polymer blend (PE/MMA_{11})

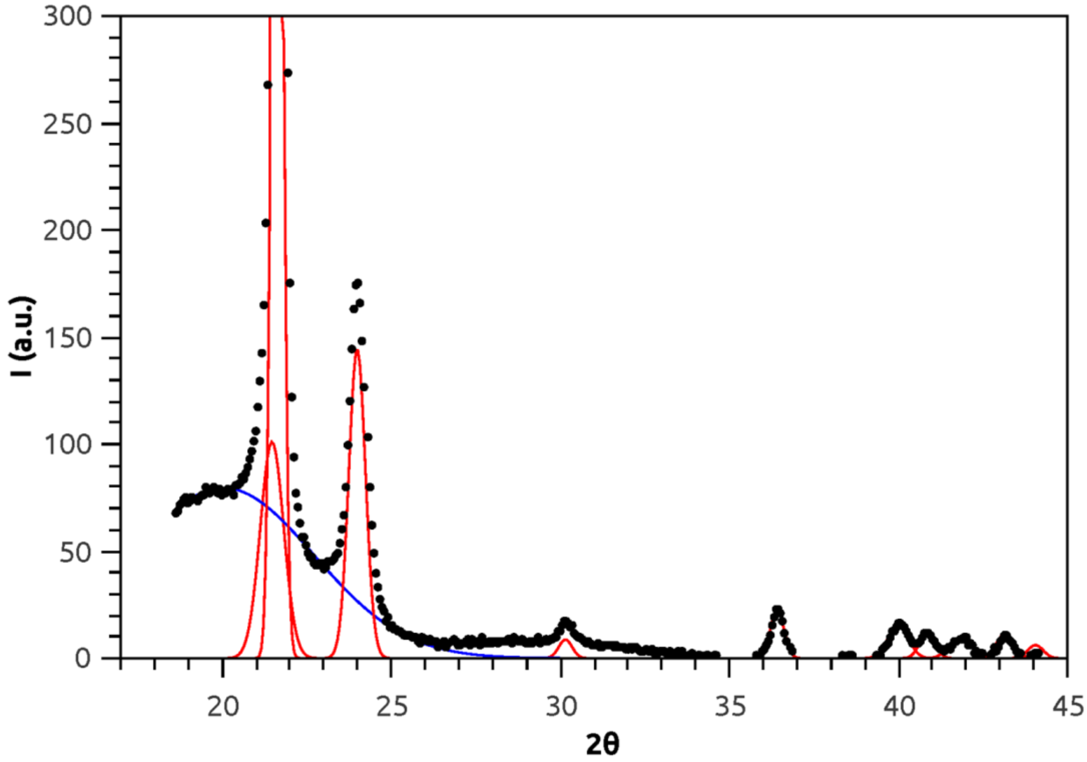

Figure S19. Diffraction pattern of PE and gaussian peak fitting of PE/MMA_{11} blend.

Table S4: Summary of the PE crystallite dimensions for PE/MMA_{11} blend. The characteristic domain size (τ) of the (110) and (011) peaks corresponds to the diameter and the thickness of the crystallite, respectively. $R = \tau_{110}$ 2 and $L = \tau_{011}$

Peak position (2θ)	Area	FWHM	τ (nm)	(hkl) orthorhombic
20.48	380.22	4.64		amorphous
21.62	307.65	0.41	79.6	110
23.99	90.60	0.59	30.5	200
30.15	8.55	0.83	17.4	210
36.42	10.66	0.45	66.1	020
40.00	16.64	0.86	18.5	011
40.86	7.12	0.50	53.8	310
41.85	15.15	1.05	14.2	111
43.14	11.37	0.68	28.4	201
44.04	7.46	0.79	22.7	220

SAXS analysis of PE-b-MMA$_{11}$ block copolymer

Here the low q-range (q < 0.02 Å^{-1}) exhibit a q^{-4} slope followed by a Guinier form factor (spheroid object).[24,25]

$I(q) \sim \exp(-Rg^2q^2/3)$

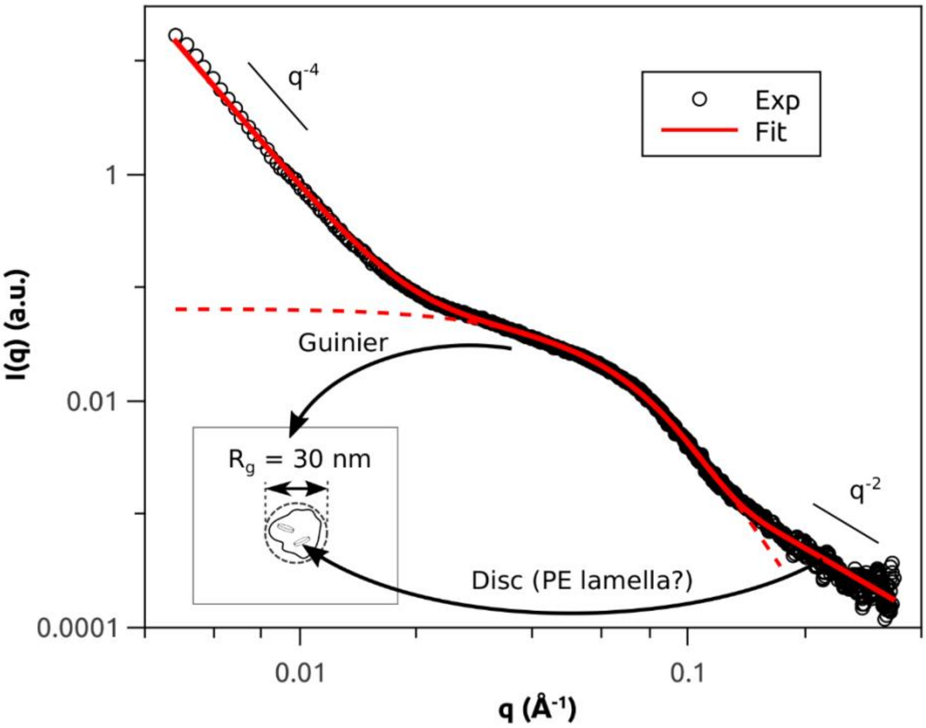

Figure S20. SAXS scattering intensities of PE-*b*-MMA$_{11}$ block copolymer.

WAXS analysis of PE/MMA$_{11}$ copolymer

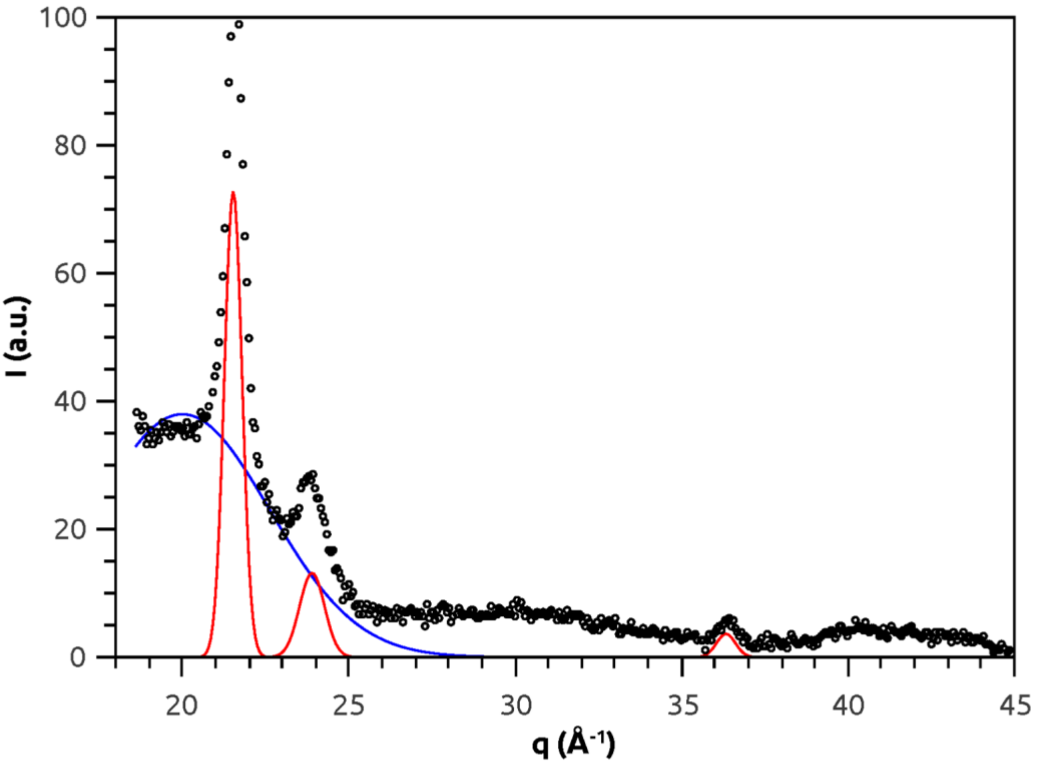

Figure S21. Diffraction pattern of PE and gaussian peak fitting of PE-*b*-MMA$_{11}$ block copolymer

Table S4: Summary of the PE crystallite dimensions for PE-*b*-MMA$_{11}$ block copolymer.

Peak position (2θ)	Area	FWHM	τ (nm)	(hkl) orthorhombic
21	229.95	5.67		amorphous
21.5	49.57	0.64	25.4	110
23.9	13.26	0.91	14.3	200

References

[1] Q. Zhang, S. Slavin, M. W. Jones, A. J. Haddleton, D. M. Haddleton, *Polym. Chem.* **2012**, *3*, 1016–1023.

[2] A. D. Becke, *J. Chem. Phys.* **1996**, *104*, 1040–1046.

[3] C. Adamo, V. Barone, *J. Chem. Phys.* **1998**, *108*, 664–675.

[4] F. Weigend, R. Ahlrichs, *Phys. Chem. Chem. Phys.* **2005**, *7*, 3297.

[5] A. V. Marenich, C. J. Cramer, D. G. Truhlar, *J. Chem. Theory Comput.* **2009**, *5*, 2447–2464.

[6] E. Mavroudakis, D. Cuccato, D. Moscatelli, *Polymers* **2015**, *7*, 1789–1819.

[7] B. B. Noble, M. L. Coote, *Int. Rev. Phys. Chem.* **2013**, *32*, 467–513.

[8] İ. Değirmenci, V. Aviyente, V. Van Speybroeck, M. Waroquier, *Macromolecules* **2009**, *42*, 3033–3041.

[9] K. H. Hopmann, *Organometallics* **2016**, *35*, 3795–3807.

[10] A. Fernández-Ramos, B. A. Ellingson, R. Meana-Pañeda, J. M. C. Marques, D. G. Truhlar, *Theor. Chem. Acc.* **2007**, *118*, 813–826.

[11] M. J. Frish, G. W. Trucks, H. B. Schlegel, G. E. Scuseria, M. A. Robb, J. R. Cheeseman, *Gaussian 09*, Gaussian, Inc.: Wallingford, CT, USA, **2009**.

[12] G. Patias, A. M. Wemyss, S. Efstathiou, J. S. Town, C. J. Atkins, A. Shegiwal, R. Whitfield, D. M. Haddleton, *Polym. Chem.* **2019**, *10*, 6447–6455.

[13] A. Shegiwal, A. M. Wemyss, M. A. J. Schellekens, J. de Bont, J. Town, E. Liarou, G. Patias, C. J. Atkins, D. M. Haddleton, *J. Polym. Sci. Part Polym. Chem.* **2019**, *57*, E1–E9.

[14] S. G. Gaynor, J.-S. Wang, K. Matyjaszewski, *Macromolecules* **1995**, *28*, 8051–8056.

[15] C. Boyer, D. Valade, P. Lacroix-Desmazes, B. Ameduri, B. Boutevin, *J. Polym. Sci. Part Polym. Chem.* **2006**, *44*, 5763–5777.

[16] A. Wolpers, F. Baffie, L. Verrieux, L. Perrin, V. Monteil, F. D'Agosto, *Angew. Chem. Int. Ed.* **2020**, *59*, 19304–19310.

[17] E. Grau, J.-P. Broyer, C. Boisson, R. Spitz, V. Monteil, *Phys. Chem. Chem. Phys.* **2010**, *12*, 11665–11669.

[18] J. Pak, B. Wunderlich, *Macromolecules* **2001**, *34*, 4492–4503.

[19] S. K. Sinha, E. B. Sirota, S. Garoff, H. B. Stanley, *Phys. Rev. B* **1988**, *38*, 2297–2311.

[20] M. Teubner, R. Strey, *J. Chem. Phys.* **1987**, *87*, 3195–3200.

[21] K. -V. Schubert, R. Strey, S. R. Kline, E. W. Kaler, *J. Chem. Phys.* **1994**, *101*, 5343–5355.

[22] J. I. Langford, A. J. C. Wilson, *J. Appl. Crystallogr.* **1978**, *11*, 102–113.

[23] C. W. Bunn, *Trans. Faraday Soc.* **1939**, *35*, 482–491.

[24] G. Porod, *Acta Phys Austriaca* **1948**, *2*, 255–292.

[25] Guinier André, Walker Christopher B, K. L. Yudowitch, *Small-Angle Scattering of X-Rays*, JWiley Chapman And Hall, New York London, **1955**.
